# 基于金属有机骨架固定相的C_8_芳香族异构体气相色谱分离研究进展

**DOI:** 10.3724/SP.J.1123.2026.01036

**Published:** 2026-11-08

**Authors:** Xuhan ZHANG, Ming XU, Zhiyuan GU

**Affiliations:** 南京师范大学化学与材料科学学院，江苏 南京 210023; School of Chemistry and Materials Science，Nanjing Normal University，Nanjing 210023，China

**Keywords:** 金属有机骨架材料, C_8_芳香族异构体, 固定相, 气相色谱, metal-organic frameworks （MOFs）, C_8_ aromatic isomers, stationary phase, gas chromatography （GC）

## Abstract

二甲苯异构体及乙基苯由于分子组成相同、理化性质高度相似，其高效分离与痕量杂质检测长期以来是色谱分离领域的经典难题。金属有机骨架材料（MOFs）凭借高度有序且可精确调控的孔道结构、丰富可设计的孔内化学环境以及多样化的主客体相互作用机制，为解决C_8_芳香族异构体分离提供了新的材料体系与解决方案。MOFs的孔径尺寸、形状各向异性及孔壁官能团可在分子尺度上进行精准调控等优点，使其在分子筛分效应、*π-π*相互作用、范德华力及扩散动力学调控等方面展现出独特优势。本文系统综述了MOFs作为气相色谱固定相在C_8_芳香族异构体分离中的研究进展。首先，总结了C_8_芳香族异构体分离的基本理论基础，重点阐述了分子筛分、孔内相互作用及扩散动力学等机制在分离过程中的作用。鉴于MOF分离过程通常涉及多种机制的协同作用，本文重点选取并讨论了各类分离机理下具有代表性的典型研究案例。随后，从不同金属节点构筑的代表性MOF材料出发，系统分析了各种MOF色谱柱的分离结果。在此基础上，进一步综述了色谱柱内壁表面修饰方法及MOF色谱柱的构筑策略，讨论其对固定相分离效果的影响。最后，总结了近年来MOF固定相分离C_8_芳香族异构体的应用和发展趋势，并对其未来发展方向进行了展望。本文旨在为高性能MOF固定相的理性设计及其在复杂芳烃体系分离中的实际应用提供参考与启示。

## Abstract (en)

The gas chromatographic separation of C_8_ aromatic isomers，including ortho-xylene （oX），meta-xylene （mX），para-xylene （pX），and ethylbenzene （EB），remains a significant and persistent challenge in analytical and separation science. This difficulty primarily arises from their identical molecular formula and extremely similar physicochemical properties. The boiling points of mX and pX differ by less than 1 K，resulting in nearly indistinguishable volatility. As a consequence，conventional gas chromatography often suffers from severe co-elution and peak overlap，which compromises both resolution and quantitative accuracy in industrial quality control. Traditional stationary phases，such as polysiloxanes and polyethylene glycols，predominantly rely on non-specific van der Waals interactions or broad polarity-based partitioning to separate analytes. These mechanisms，governed largely by thermodynamic equilibrium，often prove inadequate to amplify the subtle molecular differences among the xylene isomers. Metal-organic frameworks （MOFs） have recently emerged as a promising alternative for addressing this challenge. MOFs are crystalline porous materials constructed from metal nodes and organic linkers through coordination self-assembly. Their key advantage lies in their exceptional structural and chemical tunability. Pore size，shape，dimensionality，and internal surface chemistry can be precisely tailored at the angstrom scale，enabling rational control over separation behavior. In this review，we summarize the advantages of MOFs as stationary phases for xylene separation from three perspectives： separation mechanisms，metal-node-dependent framework design，and column fabrication strategies. First，separation mechanisms play a central role in determining the performance of MOF-based stationary phases. Owing to their exceptional design flexibility，MOFs can integrate multiple synergistic separation mechanisms within a single material. Therefore，this review highlights representative mechanistic case studies. The superior separation performance of MOFs mainly originates from three complementary mechanisms. （1） Molecular sieving and shape selectivity arising from well-defined pore apertures enable the discrimination among isomers based on kinetic diameter and molecular geometry. （2） Specific intra-pore interactions caused by different functional groups，aromatic linkers，and open metal sites on the pore walls introduce targeted non-covalent interactions，such as *π-π* stacking，hydrogen bonding，and weak coordination. These interactions magnify subtle differences in adsorption energies among C_8_ aromatic isomers，leading to the separation. （3） Kinetic mass-transfer modulation is essential for achieving high column efficiency and sharp peak shapes. This can be realized by reducing particle size to shorten diffusion paths，introducing hierarchical pore structures，or constructing two-dimensional layered architectures to regulate diffusion dynamics. Second，MOFs constructed from different metal nodes exhibit distinct separation behaviors. A wide variety of MOFs based on diverse metal centers have been explored for C_8_ aromatic separations. Zirconium-based MOFs，such as UiO-66 and PCN-608，have been extensively investigated due to their outstanding thermal and chemical stability，with separation performance further improved through morphology control and layer-stacking engineering. Aluminum-based MOFs，including MIL-160 and Al-TCPB-Me_2_，benefit from low-cost precursors and typically feature one-dimensional channels with precise size and shape recognition. Copper-based MOFs have evolved from early hydrolytically sensitive materials to ultra-stable frameworks，combining high separation resolution with long column lifetimes. In addition，titanium-，zinc-，and iron-based MOFs，such as MIL-125-NH_2_，MCF-50，and MIL-142，have also been reported to exhibit good C_8_ aromatic isomer separation capability. Third，column fabrication plays a decisive role in determining the final separation performance. This review further discusses different capillary pretreatment strategies and stationary-phase coating methods，highlighting their influence on coating uniformity，stability，and chromatographic separation performance. Finally，we provide an outlook on future development directions of MOF-based gas chromatographic stationary phases for C_8_ aromatic isomers. Future efforts should focus on improving column stability and durability，alongside developing cost-effective and scalable synthesis routes. Additionally，enhancing trace-level impurity analysis in complex matrices and facilitating integration into miniaturized and portable GC systems are crucial for practical application. This work aims to offer guidance for the rational design of high-performance MOF-based stationary phases and their practical application in the separation of complex aromatic hydrocarbons.

二甲苯是一类在常温常压下为无色、具有典型芳香性气味的液态芳烃化合物^［1］^，属于C_8_芳香族化合物体系中的重要成员。二甲苯的工业来源主要为石油重整与裂解过程所得的C_8_芳烃馏分，通常以多组分混合物形式存在。根据甲基取代基在苯环上的相对位置差异，二甲苯可以分为邻二甲苯（*o*-xylene，oX）、间二甲苯（*m*-xylene，mX）和对二甲苯（*p*-xylene，pX）。此外，乙基苯（ethylbenzene，EB）在分子组成、物理性质及工业来源上与二甲苯高度相近，在石油化工与分离领域的研究中，常将其与3种二甲苯异构体一并作为C_8_芳香族异构体体系进行讨论。尽管这些化合物在分子式和分子质量上完全相同或高度接近，但取代基的分布差异显著影响其反应行为，导致这些化合物的化学反应活性及下游应用方向存在明显区别^［2，3］^。比如，在众多C_8_芳香族化合物中，pX被认为是最具经济价值的原料之一^［4］^，是生产聚对苯二甲酸乙二醇酯（PET）的关键前体，在全球塑料和纤维工业的生产中占据核心地位。oX作为二甲苯异构体中仅次于对二甲苯的重要原料^［5］^，主要通过催化氧化转化为邻苯二甲酸酐。该产物是合成增塑剂（如邻苯二甲酸酯类）、不饱和聚酯树脂以及某些染料的重要中间体，广泛应用于建筑材料和聚合物添加剂领域。mX则主要用于合成间苯二甲酸，在PET共聚改性及高性能聚酯材料中具有独特的应用价值^［6］^。EB作为C_8_芳香族化合物中的另一重要组分，主要用于通过脱氢反应制备苯乙烯。苯乙烯是生产聚苯乙烯、丙烯腈-丁二烯-苯乙烯共聚物树脂及多种合成橡胶等高分子材料的关键前体，在塑料与橡胶工业中占据重要地位^［7，8］^。

正是由于上述C_8_芳香族异构体在下游应用中各具不可替代的工业价值^［9-11］^，其生产、储运及使用过程对组分组成与纯度提出了极为严格的要求。在实际石油化工流程中，C_8_芳烃馏分通常以多组分混合物形式存在，即使存在痕量异构体杂质，也可能显著影响后续氧化、聚合等关键反应过程，进而改变聚合物的分子质量分布及最终材料性能。因此，对C_8_芳香族异构体进行精准分离与定量分析不仅是前端化工分离过程中的核心问题，更是贯穿原料评估、过程监控及终端产品质量控制的关键环节。然而，这类化合物在分子尺寸、极性及沸点等物理化学性质上具有高度相似性，使得它们在分离分析上同样面临严峻挑战。尤其是4种C_8_芳香族异构体的沸点差异极小，在微量检测条件下极易发生共洗脱与信号重叠，严重制约了痕量杂质的准确识别与定量。这种“工业价值高度分化，分析行为高度相似”的矛盾特征，使得开发具备高分辨率与高可靠性的分析分离方法成为C_8_芳香族体系质量控制中亟须解决的关键科学与技术问题。

气相色谱（GC）因其分离效率高、灵敏度高及良好的定量准确性^［12］^，被广泛应用于石油化工产品的组成分析与质量控制。对于C_8_芳香族异构体体系而言，GC是实现原料纯度评估及痕量杂质检测的核心分析手段。然而，在实际应用中，常规气相色谱固定相多依赖非特异性的范德华作用或简单极性匹配机制进行分离。其分离行为主要受热力学分配控制，在面对结构高度相似且浓度差异显著的C_8_芳香族异构体体系时，往往难以同时兼顾分辨率、柱容量与定量可靠性，限制了其在高要求质量控制场景中的应用。在此背景下，发展新型气相色谱固定相材料以突破C_8_芳香族异构体的分离极限，逐渐成为分离科学与分析化学领域的重要研究方向。

金属有机骨架材料（metal-organic frameworks，MOFs）作为一类由金属节点与有机配体构筑的高度有序多孔晶体材料^［13-16］^，因其孔径尺寸可设计、孔道结构均一且孔内化学环境可调等特征，为精细调控气相色谱分离行为提供了新的材料平台（图1）。近年来，MOF基固定相已被成功应用于二甲苯及乙基苯的气相色谱分离^［17-20］^，不同孔径、拓扑结构及孔内微环境的MOFs在GC条件下表现出对oX、mX、pX与EB的显著选择性差异，部分体系甚至实现了4组分的基线分离和痕量杂质定量。本团队在MOF基GC固定相分离C_8_芳香族异构体领域做了大量研究工作并取得了部分研究进展。系统总结MOF固定相在C_8_芳香族异构体气相色谱分离中的研究进展，总结其分离机理、材料设计策略及存在的关键问题，对于推动该类材料在高要求分析与质量控制领域的进一步应用具有重要意义。

**图1:**
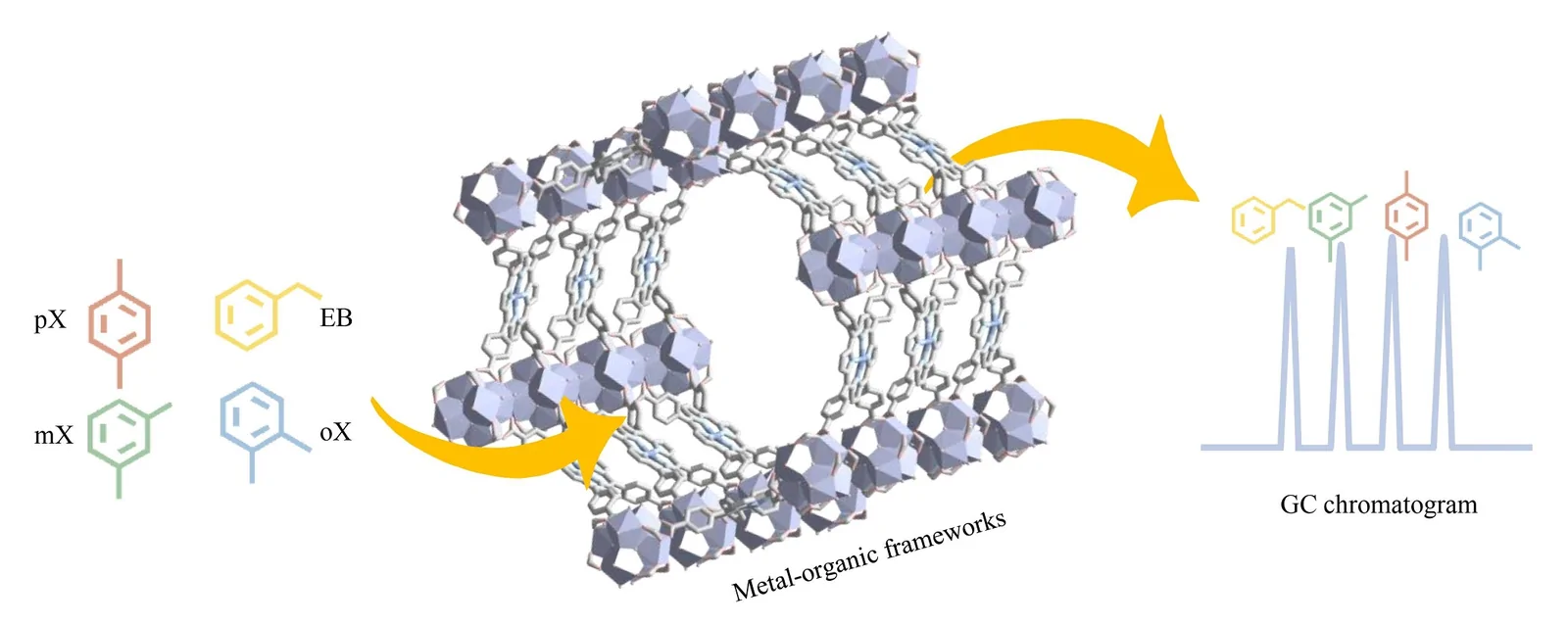
使用金属有机骨架作为色谱固定相分离4种C_8_芳香族异构体

基于此，本综述总结了MOFs材料作为固定相在C_8_芳香族异构体分离中的最新进展，重点围绕分离机理、代表性材料体系及结构调控策略进行讨论，并对其在实际分析与质量控制中的应用前景进行展望。

## 1 C_8_芳香族异构体的分离机理

深入理解C_8_芳香族异构体在MOFs中的分离机理，是实现其高选择性分离与精准分析的重要基础。作为石油化工体系中结构高度相似的一类芳香族异构体，C_8_芳香族异构体在分析尺度上的分离行为往往受到分子识别能力有限与相互作用差异微弱的双重制约。在气相色谱分离过程中，异构体的保留与洗脱不仅取决于热力学分配特性，还受到分子在固定相孔道中的传质行为及界面相互作用的显著影响。因此，从分子尺度系统阐明C_8_芳香族异构体的分离机理，对于理解固定相结构与色谱性能之间的内在关联具有重要意义。MOFs作为气相色谱固定相材料，其对二甲苯及乙基苯的分离通常并非由单一作用机制主导，而是多种因素协同作用的结果。现有研究普遍认为，MOF固定相中C_8_芳香族异构体的分离机理主要体现在以下几个方面：（a）分子筛分与形状选择机制，（b）孔内相互作用差异机制，（c）动力学传质调控机制。

### 1.1 分子筛分与形状选择机制

分子筛分原理是指多孔材料依托其均一且固定的孔径尺寸及孔道构型，对不同分子产生选择性进入与传输限制，从而实现分离的一种机制^［21］^（图2a）。对于C_8_芳香族异构体体系而言，尽管oX、mX、pX与EB在分子结构上高度相似，但是其动力学直径存在微小差异（表1），因此可以通过设计具有特定孔径和孔道结构的材料，利用孔道尺寸的筛分效应，实现4种异构体的分离。其中，pX在二甲苯异构体中具有最小的动力学直径，更容易进入受限的孔道，因此分子筛分在pX的分离中尤为显著。Yonezawa等^［22］^制备了一种MOF-801微孔锆基金属有机骨架材料，该材料的微孔尺寸会随温度的变化而变化。实验表明，低温时，二甲苯异构体无法进入孔道中，会迅速从色谱柱上洗脱。当温度升高时，MOF-801会对二甲苯分离表现出分子筛效应（图2c）。其中，oX和mX因动力学直径较大无法进入微孔，快速流过颗粒间隙，而对二甲苯可扩散进入微孔并延迟洗脱，从而实现选择性分离。利用分子筛分机制，本团队^［23］^报道了一种名为NU-901的微孔MOF色谱固定相，它可以从邻、间、对3种二甲苯异构体混合物中选择性地分离pX。NU-901纳米颗粒具有小于0.7 nm的菱形通道。将该材料涂覆在毛细管柱上用于分离二甲苯异构体时，具有最小动力学直径的pX更容易进入NU-901的孔道中。因此，pX/mX和pX/oX的分离度（*R*_s_）值都远远大于mX/oX的*R*_s_值。除了pX对分子筛分策略表现出高度敏感性之外，oX由于分子横向尺寸较大，其扩散与进入微孔通道同样受到显著的分子筛限制作用，因此分子筛效应在oX的分离行为中也起着重要作用。比如，最近本团队^［24］^设计了一种具有不对称弯曲一维孔道的铝基金属-有机骨架材料（Al-TCPB-Me_2_，TCPB-Me_2_=1，2，4，5-四（4-羧基苯基）-3，6-二甲基苯）。该材料的孔径与oX和mX的动力学直径接近。但是，与接近线性的mX相比，oX的邻位双甲基在孔道入口处受到更显著的位阻，因此难以进入孔道中。在分离时，oX最先被洗脱。这一结果与其在常规商用色谱柱上的行为形成鲜明对比。oX具有4种C_8_芳烃异构体中相对最高的沸点，通常表现为最晚出峰。本团队所设计的分离材料利用分子筛策略使得oX首先被排除，打破了沸点主导的洗脱序列，为结构高度相似异构体的精准分离提供了一种全新的调控思路。

**图2:**
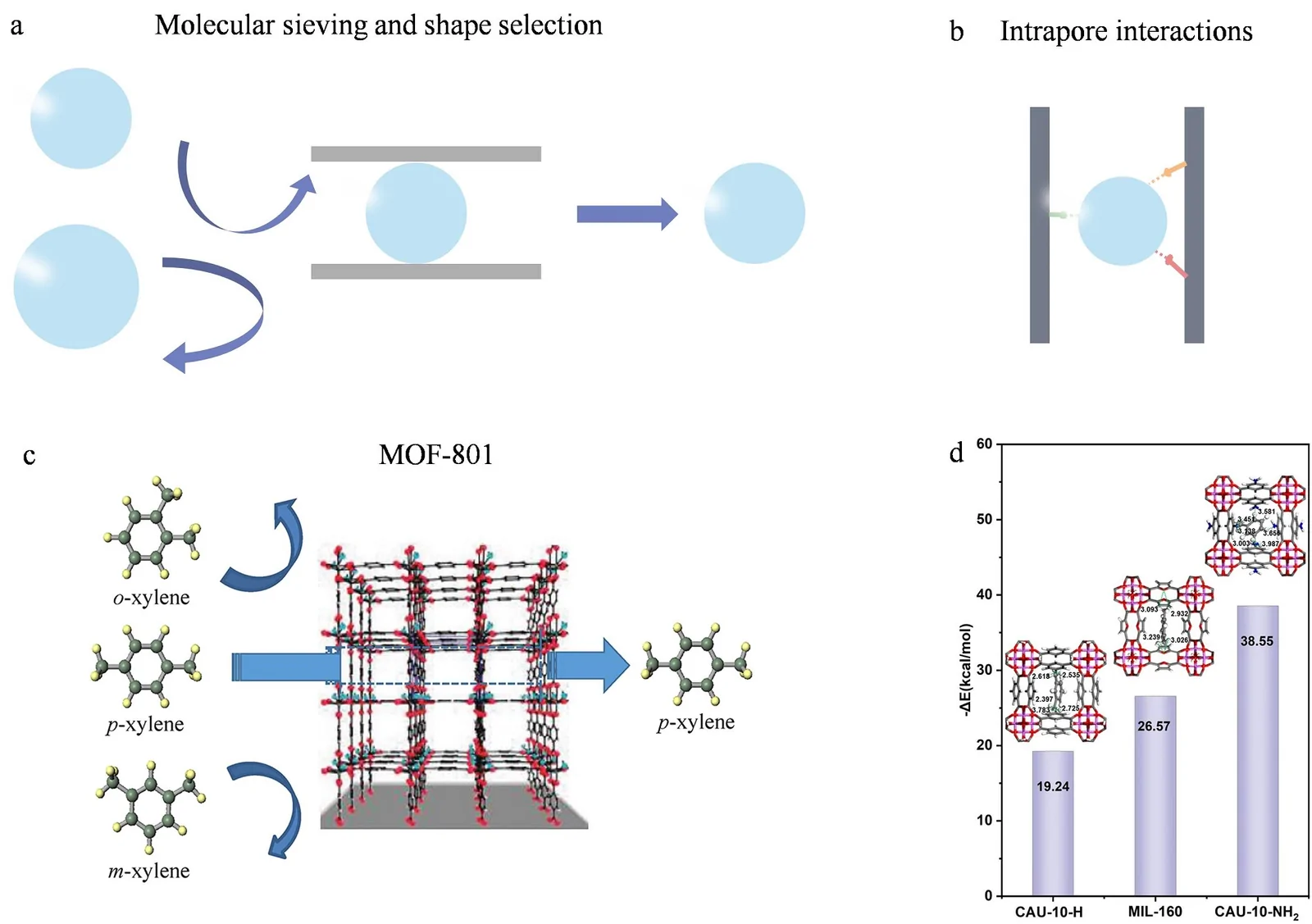
（a）分子筛分效应示意图和（b）孔内相互作用示意图；（c）使用MOF-801作为色谱固定相分离3种二甲苯异构体示意图^［22］^；（d）mX在铝基金属有机骨架中的吸附能^［25］^

**表1:** 4种C_8_芳香族异构体的动力学直径及沸点

Xylene isomer	Kinetic diameter/nm	Boiling point/℃
*p*-Xylene	0.58	138.4
*m*-Xylene	0.68	139.2
*o*-Xylene	0.68	144.4
Ethylbenzene	0.58	136.2

形状选择性是指多孔材料在孔径尺寸相近甚至相同的条件下，依托孔道几何形态、弯曲程度及局域化学环境等结构特征，对分子构型、取代基分布和空间取向产生差异化限制，从而实现选择性吸附与传输的一种分离机制。与以孔径大小为主导的分子筛分效应不同，形状选择性更强调孔道与分子三维构型之间的匹配程度，尤其在孔径接近分子临界尺寸时，对异构体分离具有重要作用。对于二甲苯异构体体系而言，尽管oX、mX和pX的动力学直径差异有限，但其取代基位置与分子整体构型存在显著差别，使其在弯曲、不对称或具有局域位阻的孔道中表现出不同的进入能力与扩散行为。因此，我们团队通过实验研究结合理论模拟发现，通过精确调控孔道的几何形貌和构型约束，可在尺寸筛分作用之外进一步放大异构体之间的分离差异，为实现高选择性分离提供新的设计维度。基于上述分离机理，本团队^［24］^设计的Al-TCPB-Me_2_材料利用分子筛分策略排除oX，同时使mX和pX共同进入孔道。由于pX具有更大的纵向尺寸，其分子构型与弯曲孔道的几何形态更加匹配，在孔道内受到更强的构型约束，从而表现出更强的保留和更晚的洗脱。受分子筛分效应与形状选择协同作用的影响，Al-TCPB-Me_2_对二甲苯异构体表现出优异的分离性能。其中，mX/pX的*R*_s_高达24.9，oX/pX的*R*_s_进一步提升至31.1，远高于先前报道的大部分固定相材料，充分体现了该材料在尺寸与构型双重约束下对异构体的高效区分能力。

基于以上研究可以总结认为，分子筛分效应通过孔径限域对分析物进入与传输进行“准入控制”，能够显著放大单一目标物与其余组分之间的保留差异，既可大幅提升特定异构体与其他组分之间的分离度，也可在特定条件下改变传统由沸点或整体亲和性主导的洗脱顺序；而形状选择性则在孔径接近分子临界尺寸时进一步发挥作用，通过孔道几何构型与分子空间构型之间的匹配或失配，持续放大结构高度相似异构体之间的细微差异，从而实现精细分离。当分子筛分效应与形状选择性协同引入同一分离体系时，可在“准入筛选-孔道内构型识别”两个层级同时施加约束，显著提升分离度，为复杂异构体体系的精准分离提供了一种高效且可设计的结构调控策略。

### 1.2 孔内相互作用差异机制

孔内相互作用差异性是指通过精准调控多孔材料孔壁的化学性质、官能团类型及其空间取向，在孔内构建差异化的相互作用环境，从而对不同分子在吸附强度、扩散动力学和立体识别行为上产生选择性调控的一种分离机制。其核心在于利用孔环境的结构与化学特性，对目标分子的几何构型、官能团分布及动力学响应进行区分性作用，进而放大结构相似分子之间的微小差异，实现高效识别与分离（图2b）。基于以上机理，本团队^［25］^选取了3种具有相同fym拓扑结构的铝基MOF（CAU-10-H、CAU-10-NH_2_与MIL-160），通过改变V型配体的种类（分别对应为间苯二甲酸、5-氨基间苯二甲酸与2，5-呋喃二甲酸），在埃米尺度上精细调节了孔道尺寸与化学环境。CAU-10-H的中性苯环孔壁仅提供较弱的范德华作用（图2d），难以对不同二甲苯异构体形成有效保留，因而分离选择性较低。相比之下，MIL-160中呋喃环上的氧原子引入了更强的极性作用与氢键位点，在不显著阻碍分子扩散的前提下适度增强了分析物在孔道内的有效保留，强化了位点对分析物的识别作用。因此，MIL-160可实现对二甲苯异构体的高效分离。mX/pX、oX/pX和mX/oX的*R*_s_分别为16.28、19.00和4.55，超越了多数已报道的材料。为进一步加强热力学作用力，本团队合成了CAU-10-NH_2_。该材料中的氨基导致了过强的热力学作用与明显的空间位阻，使得分析物在孔道中扩散过慢，从而造成色谱峰展宽，分离性能下降。在此基础上，本团队^［26］^又从增强热力学差异化的角度进一步证实了孔内相互作用差异在二甲苯异构体分离中的重要作用。通过在MIL-125中引入氨基官能团，成功合成了MIL-125-NH_2_。研究表明，氨基的引入可通过N-H…*π*等特异性相互作用显著增强对芳香环分析物的差异性吸附，使MIL-125-NH_2_对二甲苯异构体的分离能力优于MIL-125。随后，本团队^［26］^又在MIL-143这一体系中，将以苯为核心的配体替换为以三嗪为核心的配体，构建了MIL-143-TATB（TATB=2，4，6-三（4-羧基苯基）-1，3，5-三嗪）。该材料具有较强的电子亲和性与多重作用位点，可以对二甲苯异构体产生显著差异化的吸附，实现二甲苯异构体的高效分离。而结构相同、但以苯为核心的MIL-143-BTB（BTB=1，3，5-三（4-羧基苯基）苯）则因缺乏这类特异性相互作用，几乎不具备分离能力。因此，通过对MOF孔壁官能团类型、极性分布及空间取向的精准调控，在孔内构建差异化相互作用环境，即形成“孔内相互作用差异化调控机制”，可有效放大二甲苯异构体及乙基苯之间的热力学差异，是突破结构高度相似分子分离瓶颈的一条关键路径。

### 1.3 动力学传质调控机制

动力学传质调控机制是指在色谱分离过程中通过调控分析物在固定相中的扩散速率与传质阻力，以优化其吸附-解吸平衡与迁移行为，从而实现高效分离的策略。该机制的核心在于对传质过程的主动干预，以解决因扩散过程不匹配而导致的峰展宽、峰拖尾或分离度不足等问题（图3）。值得一提的是，本团队率先开始针对分析物在MOF固定相中动力学行为的精细化研究。早期相关研究多停留在宏观色谱性能表征层面，主要通过理论塔板数、等板高度以及传质阻力项对分离效率进行定性或半定量评估，而对分析物在固定相内部的真实扩散行为缺乏直接刻画。本团队进一步参考戈雷（Golay）方程，从色谱动力学出发定量计算分析物在MOF固定相中的有效扩散系数，首次将扩散过程作为可调控的关键变量，系统揭示其对峰形演化与分离选择性的影响。基于这一认识，本团队分别从“加速传质”和“延缓传质”两个相反但互补的调控方向出发，通过精准设计孔道尺寸、形貌及相互作用位点，构建具有可编程动力学特征的固定相材料，从而实现对复杂体系中相似组分的高效分离。

**图3:**
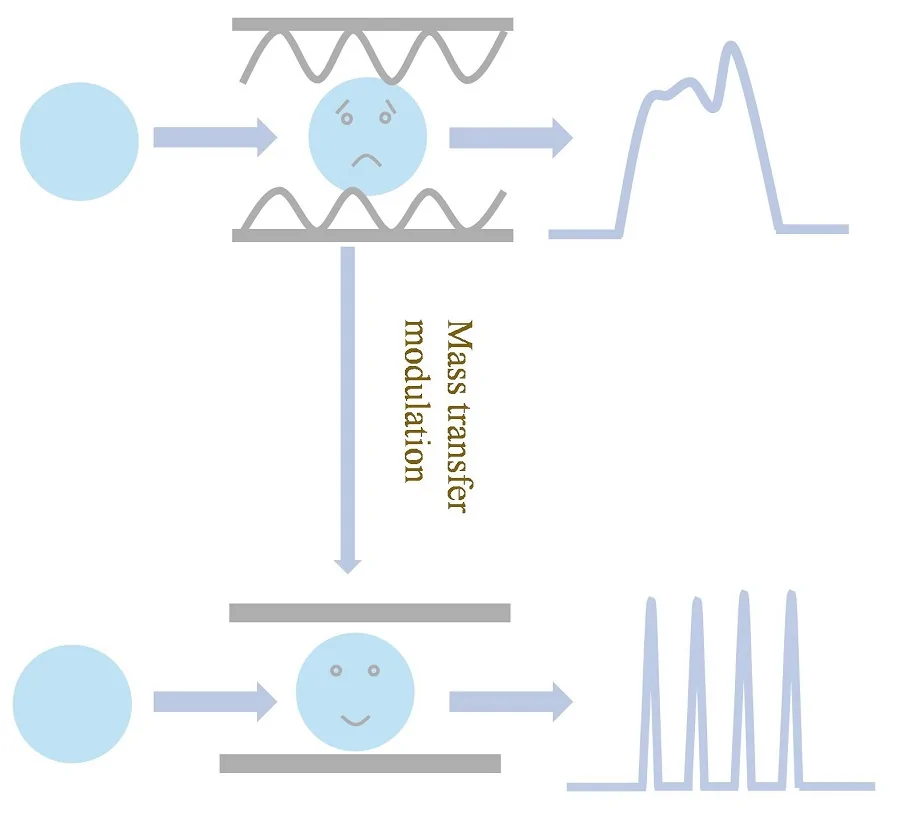
动力学传质调控示意图

从加快传质角度，本团队^［27］^提出了尺寸控制的方法将微米级的色谱固定相（NU-1000、PCN-608和PCN-222）缩小至纳米尺度，显著缩短分析物在固定相内的扩散路径、减少堆积孔的形成，从而降低传质阻力、提高柱效，验证了通过加速传质以改善分离性能的可行性。从延缓传质的角度，本团队^［28］^针对因孔隙过大、传质过快而导致分析物保留过弱、难以达到吸附-解吸平衡的MOF固定相，提出了“逆向调控”策略。以UiO-67为例，通过将三维孔道结构转换为二维纳米片结构（图4a），缩小有效孔道尺寸，从而增加传质阻力、延长分析物在固定相中的停留时间。实验表明，二维UiO-67纳米片相比其三维结构表现出更低的扩散系数。这主要是由于二维UiO-67缺少了三维结构中两个大于1.0 nm的孔腔，仅保留了约0.8 nm的孔径窗口，使分析物在孔道内的迁移受限，从而显著减缓其扩散速度，延长在孔道中的滞留时间。二维UiO-67对异构体的分离度显著优于三维UiO-67（图4b），说明在传质过快的情况下，适当增加传质阻力同样可优化分离效率。基于上述研究结果，本团队得到结论，单纯地加速或抑制传质并不能普遍保证分离性能的提升，适中的传质速率才是实现高效分离的最优状态。过快的传质会削弱分析物在固定相中的选择性相互作用，而过慢的传质则易引发显著的峰展宽和分析时间延长。因此，后续研究聚焦于对传质过程的精细调控，以获得在分离度、柱效与分析效率之间高度协同的色谱性能。本团队^［29］^提出可以通过在单个纳米晶内均匀混合具有微孔结构的NU‑901与具有介孔结构的NU‑1000来制备MOF固溶体，从而构建了微孔‑介孔比例连续可调的复合材料体系。此设计使材料兼具微孔的强吸附作用与介孔的快速扩散通道，从而精准调节分析物的传质速率。当微孔与介孔比例适当时，材料在分离二甲苯等异构体时展现出最优性能，有效避免了因传质过慢导致的峰展宽与因过快导致的保留不足，实现了传质动力学的“平衡态”调控。随后，本团队^［30］^又通过调控不同长度配体（对苯二甲酸（BDC）与2，6-萘二羧酸（NDC））的比例，构建了一系列孔径连续可调的MOFs材料（CTMOF-1~3）。实验表明，随着长配体比例增加，材料孔径逐渐增大，传质速率相应提升；然而分离性能并未随传质加速而单调提高。传质过慢（CTMOF-1）会导致严重峰展宽，传质过快（CTMOF-3）则因作用时间不足而使分离度下降。唯有在传质速率适中的CTMOF-2中，材料才展现出最高的分离度。

**图4:**
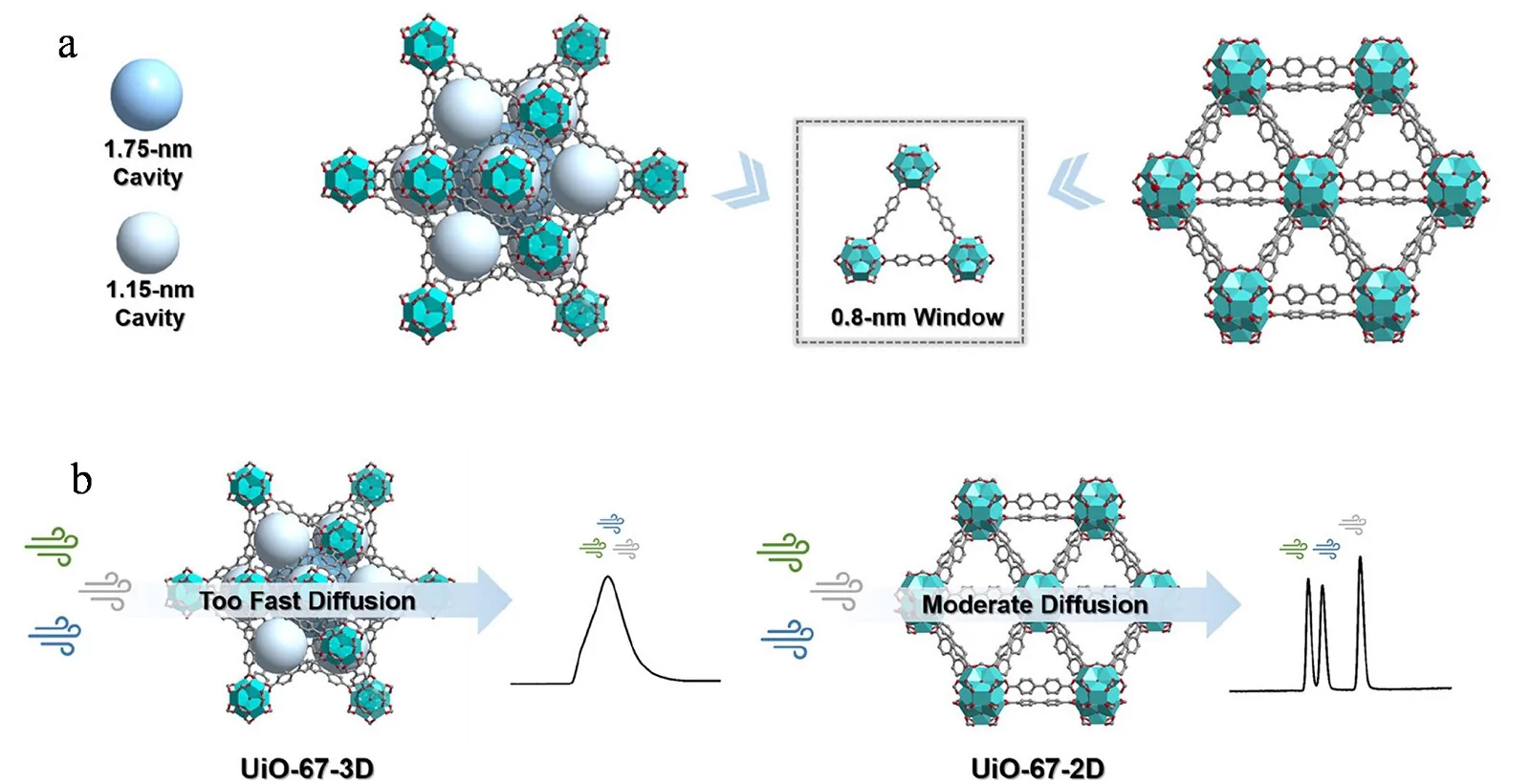
（a）UiO-67-3D与UiO-67-2D的结构对比图和（b）分析物在UiO-67-3D与UiO-67-2D中的传质速率示意图^［28］^

综上，本团队认为C_8_芳香族异构体在MOF固定相中的分离，本质上是热力学相互作用与动力学传质之间的竞争与平衡。过去的研究往往侧重于强化孔道吸附能力或热力学选择性（如增加孔壁范德华力、引入极性官能团），却容易忽视扩散受阻带来的峰形拖尾与传质瓶颈。通过系统的对比实验，本团队发现对于孔径在0.5~0.8 nm（与二甲苯动力学直径相近）范围内的MOF，传质阻力是限制分离度提升的关键因素之一。由此可见，未来高性能MOF固定相的设计不应单纯追求极高吸附能，而是应结合考虑形貌、孔比例等各种因素。在保持热力学选择性的同时显著降低传质阻力，从而实现结构高度相似异构体的高效分离。

## 2 已用于分离C_8_芳香族异构体的MOF固定相分类

自Finsy等^［20］^首次在气相色谱领域使用MOFs填充柱分离C_8_烷基芳烃异构体之后，MOFs材料凭借其高度可调的孔道尺寸、丰富的表面化学特性以及优异的结构稳定性，在色谱分离领域展现出显著优势。研究人员相继开发了多种基于MOFs的色谱固定相（图5），相关研究数量呈现出迅速增长的趋势^［31-35］^。鉴于MOFs的结构与性能在很大程度上受金属节点类型的影响，后续研究常以金属中心为分类依据，对不同MOF固定相的色谱分离行为进行系统比较。因此，以下将按照金属节点类型对已报道的用于C_8_芳香族异构体分离的典型MOF固定相进行归纳与讨论。

**图5:**
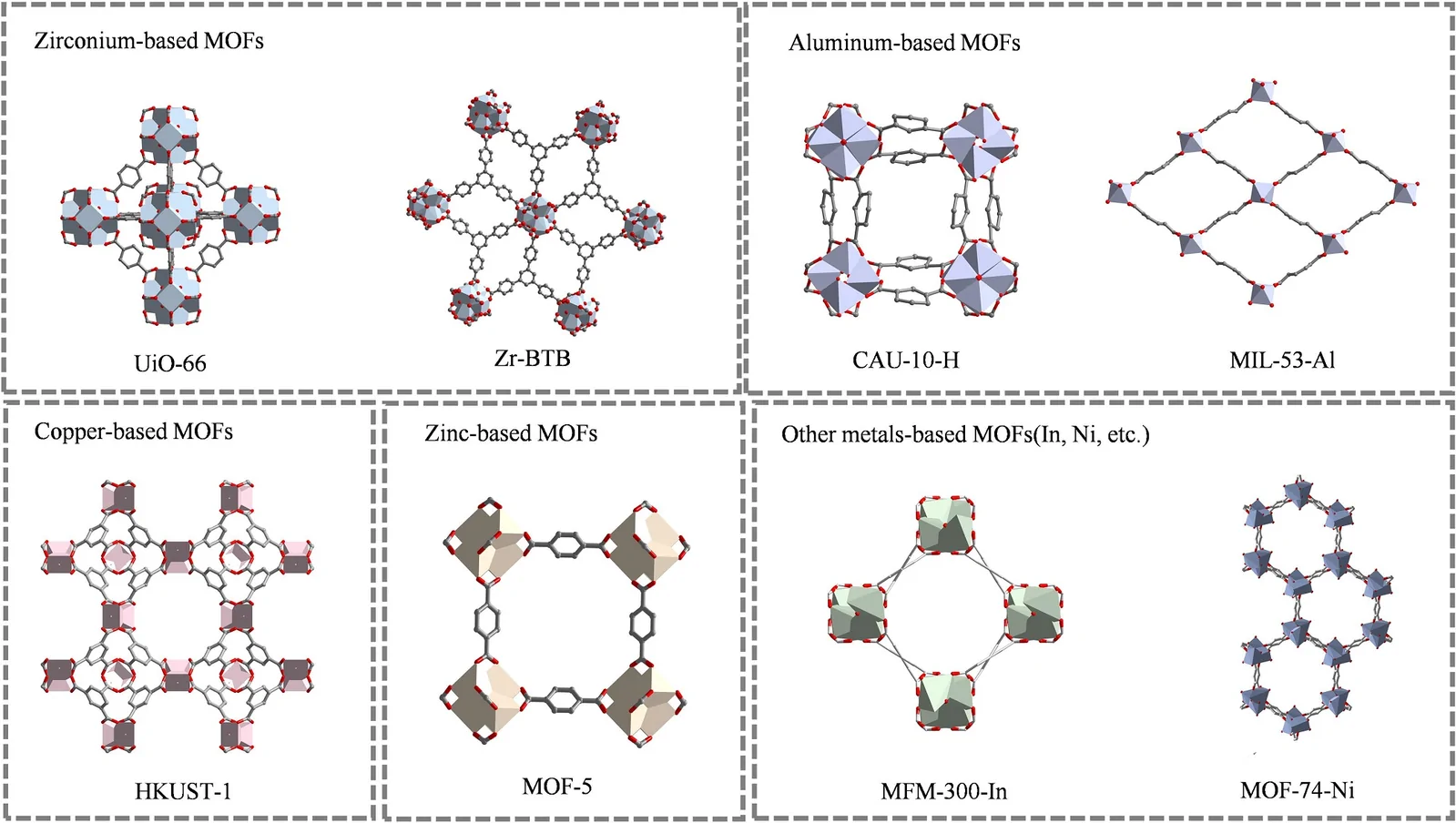
典型金属有机骨架结构示意图

### 2.1 锆基MOF固定相

2008年，锆基MOF（UiO-66）首次合成，其凭借高化学和热稳定性、大比表面积以及小孔径等优点成为科学界的研究热点^［36-39］^。Zr-MOFs以锆氧簇（如Zr_6_簇）和羧酸为次级结构单元（SBUs），硬酸硬碱形成的强配位键赋予其卓越的化学与热稳定性。通过调节有机配体的几何构型与官能团，可精准调控材料的拓扑结构，实现从微孔至介孔的精确调节与表面化学修饰。正是基于上述稳定性与结构可设计性的综合优势，本团队早期选择将锆基MOF作为气相色谱固定相的研究对象，系统开展了其合成、结构调控及分离性能研究，并在此基础上持续发表了一系列相关工作。在色谱分离领域，Zr-MOFs不仅可以通过尺寸筛分与各种相互作用实现对二甲苯等异构体的高选择性分离，还可通过粒径纳米化、形貌调控、堆积调控及层间褶皱工程等策略优化传质动力学，提升柱效并抑制峰拖尾。因此，锆基MOFs是气相色谱固定相的理想材料。

UiO-66是通过将六核锆簇（Zr_6_O_4_（OH）_4_）与桥接配体BDC连接而成的三维多孔结构（图6a）^［40，41］^。Yan等^［42］^用UiO-66涂覆毛细管色谱柱，系统考察了其对二甲苯异构体的分离性能。UiO-66拥有约为0.7 nm的孔径，在分离中表现出对oX的优先选择性。oX的保留时间长于mX、pX和EB。这种选择性主要源于oX的分子尺寸（最大直径约0.74 nm）与UiO-66的孔径相匹配，oX能够进入孔道内部，并与孔壁产生较强的范德华相互作用。此外，oX极性较高（偶极矩为0.54 D），其偶极可诱导产生氢键作用，进而与材料中酸性的锆位点发生特异性相互作用，进一步强化了保留行为。尽管UiO-66在分离二甲苯异构体时表现出明显的选择性，但其柱效并不理想，峰形展宽较为明显（图6c）。为了提升UiO-66对二甲苯的分离能力，本团队对UiO-66的结构进行整体分析，推测可能是UiO-66的孔径窗口较小，分析物在受限空间内的传质阻力较大，导致了色谱峰的拖尾。为此，本团队^［30］^在UiO-66的合成过程中混入更长的NDC配体，通过调控配体BDC和NDC的比例，合成了一系列孔道尺寸渐变的连续可调的CTMOFs材料，以精确优化传质过程。研究证实，具有适中传质速率的CTMOF-2对于pX与oX的分离度高达5.96，远优于常规商业柱及其他MOFs材料，并实现了丙烯与丙烷的高效气相色谱分离。

**图6:**
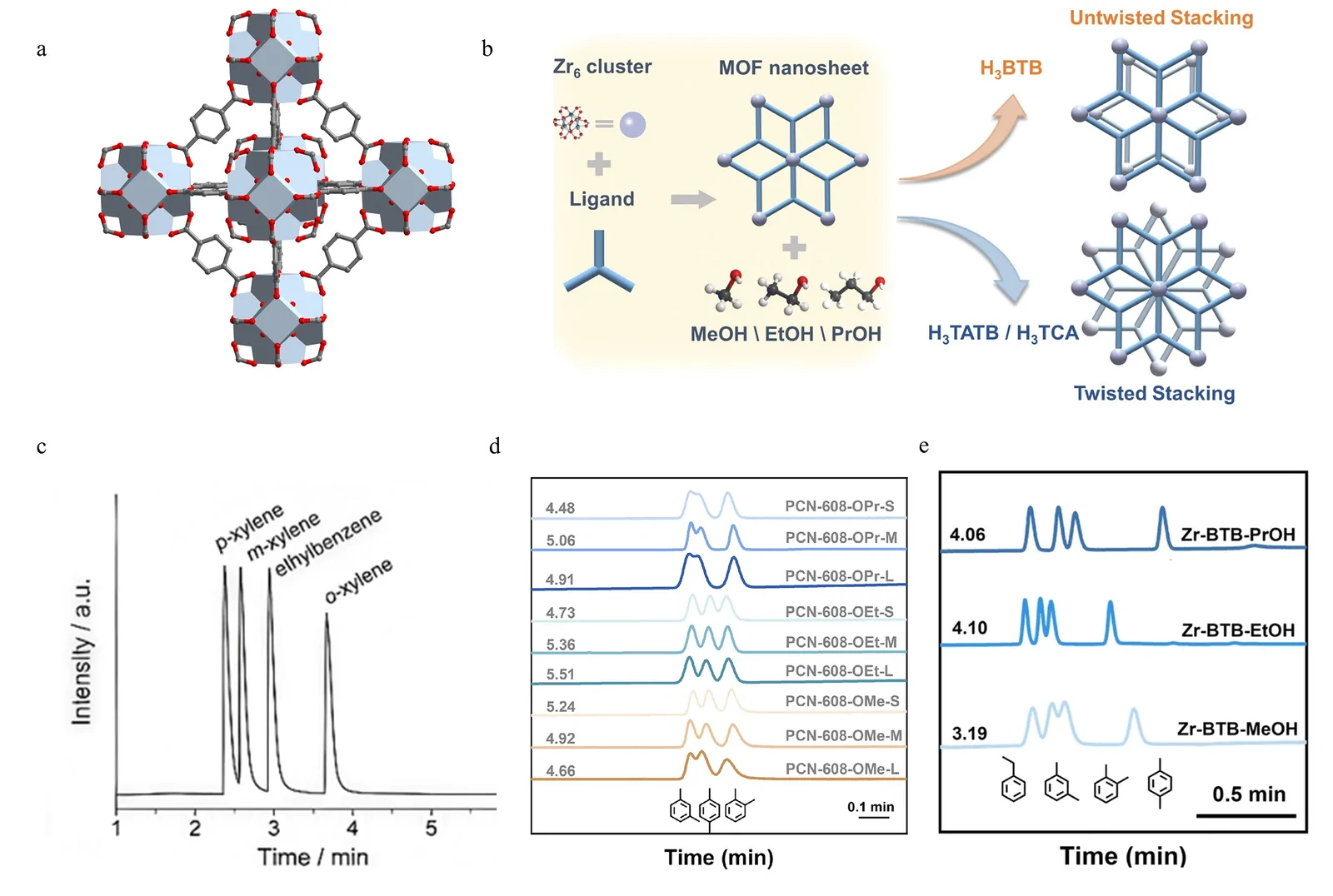
（a）UiO-66结构示意图和（b）通过极性醇类客体分子调控二维Zr-BTB纳米片堆叠方式的示意图^［49］^；（c）UiO-66、（d）PCN-608-OMe、PCN-608-OEt及PCN-608-OPr系列和（e）二维Zr-BTB纳米色谱柱的气相色谱图^［42，44，49］^

作为锆基MOF固定相的另一类代表性材料，PCN-608系列在色谱分离中日益受到关注。PCN-608作为一种具有CSQ拓扑和分级孔结构的锆基MOF，其固有的刚性骨架、可调控的孔径以及富含芳香环的连接体，为C_8_芳香族异构体的高效色谱分离提供了理想平台。该材料具有介孔与微孔并存的多级孔道，可系统实现对各种异构体的尺寸筛分与形状识别，同时配体上的芳香环还可以与C_8_芳香族异构体分子产生特异性*π-π*相互作用，在热力学上增强对异构体的吸附选择性。基于PCN-608的结构优势，本团队^［27］^合成了一种纳米级PCN-608，并将其制备成GC色谱柱。尽管该材料在烷烃异构体的分离上表现出了优异的分离性能，但其对C_8_芳香族异构体的分离效果不佳。在此基础上，本团队^［43］^提出了进一步的优化策略。通过“配体剪切”策略精准设计了双齿封端调制剂，诱导PCN-608晶体沿特定晶面方向生长，实现了PCN-608从纳米片到纳米棒形貌的可控构筑。PCN-608纳米片一维通道的孔壁虽能提供*π-π*相互作用，但因该作用力强度有限，无法有效区分C_8_芳香族异构体间的微小差异。将材料形貌调控成较长的纳米棒以后，物理上延长一维孔道的长度，增加分析物在孔道中的滞留时间，强化孔道对不同分析物的识别作用，提升了PCN-608的分离度。更进一步地，本团队^［44］^继续优化PCN-608的分离性能。将PCN-608原有配体上的甲氧基（OMe）更换为乙氧基（OEt）和丙氧基（OPr），发现PCN-608-OEt具有适中的作用力和传质阻力，可以实现对二甲苯异构体的基线分离（图6d）。

Zr-BTB作为一种典型的二维锆基金属有机骨架纳米片，是由六核锆簇与平面三齿羧酸配体BTB通过配位键连接而形成的一类多孔材料，单片层的孔径约0.88 nm。与UiO-66和PCN-608等锆基MOF类似，Zr-BTB同样表现出优异的化学稳定性和热稳定性。本团队在早期将Zr-BTB引入气相色谱固定相研究时发现，其分离行为与传统三维MOF固定相存在显著差异。得益于其二维片层结构特征，Zr-BTB可通过调控层间扭转角度实现有序堆叠，从而形成具有亚埃级可调孔径的堆积孔道。该结构特征不仅能够有效调控分析物在孔道中的传质速率，还可增强对异构体的差异化吸附行为。本团队^［47］^利用客体溶剂分子的主客体非共价相互作用来精准调控Zr-BTB纳米片的堆叠模式。研究表明，弱极性的甲苯与乙酸乙酯可以诱导纳米片产生具有特定扭转角（如6°、12°、18°等）的扭曲堆叠；而非极性的烷烃分子则因空间位阻及疏水作用垂直进入孔道，导致纳米片呈现非扭曲堆叠，形成高度有序的层间纳米通道。作为色谱固定相，非扭曲堆叠的Zr-BTB样品展现出对C_8_芳香族异构体等苯系异构体的优异分离能力，尤其是对mX和pX的*R*_s_约为3.27，显著优于扭曲堆叠样品及部分商用色谱柱。该工作首次证明利用客体分子调控二维MOF的堆叠模式，是构筑具有高效气相色谱分离性能有序孔道的关键策略。随后，本团队^［49］^又将客体调控策略拓展至极性醇类分子（甲醇、乙醇、丙醇），系统研究了极性作用对堆叠行为的影响（图6b）。与上述弱极性的甲苯与乙酸乙酯不同，尽管醇类具有较强的极性，其诱导的Zr-BTB纳米片却呈现高比例的非扭曲堆叠（占比77%~86%）。理论计算表明，醇分子的羟基与Zr_6_簇上的开放位点发生强极性相互作用，促使相邻层间趋向有序排列。所得非扭曲堆叠的Zr-BTB固定相对C_8_芳香族异构体表现出高分辨率、良好的对位选择性与优异的柱稳定性（图6e）。该研究揭示了极性客体分子与MOF表面位点的特异性相互作用是实现堆叠精准调控的新机制。进一步研究发现，当Zr-BTB纳米片的厚度降低至足够薄的尺度时，其二维片层表面会自发形成褶皱结构。这些褶皱并非简单的形貌缺陷，而是在纳米尺度上构筑了额外的扩散通道，为分析物在固定相中的迁移提供了新的扩散路径。不同褶皱形态在曲率、宽度及连续性方面的差异，会显著改变分析物的有效扩散距离与扩散阻力，从而调控其在固定相中的扩散动力学行为，并最终反映为分离度的变化。基于此，本团队^［50］^提出可以通过合成时加入的羧酸调节剂的链长来系统调控Zr-BTB纳米片的形貌，创新性地获得了具有不同程度褶皱的超薄纳米片。本团队选用甲酸至辛酸等一系列羧酸作为调节剂，合成了具有不同亲疏水性的Zr-BTB-C_x_（C_x_=甲酸到辛酸）纳米片。研究发现，随着烷基链增长，MOF层间相互作用增强，而MOF-溶剂相互作用减弱，二者协同作用诱导了纳米褶皱的形成。其中，丁酸调控合成的Zr-BTB-C_4_呈现出最显著的褶皱结构，其层间形成的介孔通道（约3.98 nm）有效促进了分析物的传质，降低了色谱峰的拖尾。作为气相色谱固定相，Zr-BTB-C_4_在分离二甲苯、氯甲苯及二氯苯等异构体时都展现出优异的分离度，显著优于无褶皱或褶皱程度较低的样品。该研究详细揭示并证明了通过精细调控二维MOF纳米片的褶皱形态，也可优化纳米片整体的孔道结构，从而提升固定相的色谱分离性能。

### 2.2 钛基MOF固定相

钛基金属有机骨架材料（Ti-MOFs）因其优异的热稳定性而成为一类重要的气相色谱固定相。其中，MIL-125（Ti）是最具代表性的Ti-MOFs材料之一。该材料以由羟基五氧合钛八面体构成的环状八聚体作为金属节点，并通过BDC配体桥连组装而成。其结构特征主要表现为独特的双笼系统（图7a），这种双笼系统在色谱分离中发挥了协同增效的关键作用。开放的四面体笼（0.6 nm）因其尺寸较小可以发挥分子筛效应，能基于分子临界直径与形状对异构体进行初步区分，允许细长构型的分子（如pX）优先进入并快速扩散，而将体积更大或形状不匹配的分子（如oX和mX）部分阻隔在外。较大的八面体笼（1.2 nm）则作为主要的吸附、分离与传质场所，可以为进入的分析物提供充分的空间以发生有效的热力学相互作用，包括范德华力、极性作用以及可能的氢键，从而根据分子与孔壁作用强度的差异实现进一步的精细分离。2016年，Van der Perre等^［51］^报道了采用单分散MIL-125（Ti）晶体作为高效液相色谱固定相的研究。在乙腈流动相中，MIL-125（Ti）固定相对二甲苯异构体表现出明确的对位选择性，对不同组分之间的选择因子为1.1~2.1。二甲苯的洗脱顺序（邻位<间位<对位）与分子尺寸变化趋势一致。这种选择性源于其开放四面体笼对尺寸较小的pX的优先吸附。基于MIL-125（Ti）的结构优点，本团队^［26］^以MIL-125（Ti）作为研究对象，并通过在MIL-125的有机配体中引入氨基官能团，合成了MIL-125-NH_2_。然后将这两种材料都制备成了GC固定相。MIL-125-NH_2_相比于MIL-125保留了原有的双笼结构，同时氨基的引入显著增强了孔壁与目标分析物之间的相互作用，提升了材料的分析物识别能力（图7b）。在气相色谱分离中，MIL-125-NH_2_涂覆的毛细管柱对C_8_芳香族异构体表现出优异的分离能力，其分离度显著高于未修饰的MIL-125（图7c）。其中MIL-125-NH_2_对pX和mX的分离度达到13.10（图7d），而MIL-125对pX和mX的分离度为6.90。

**图7:**
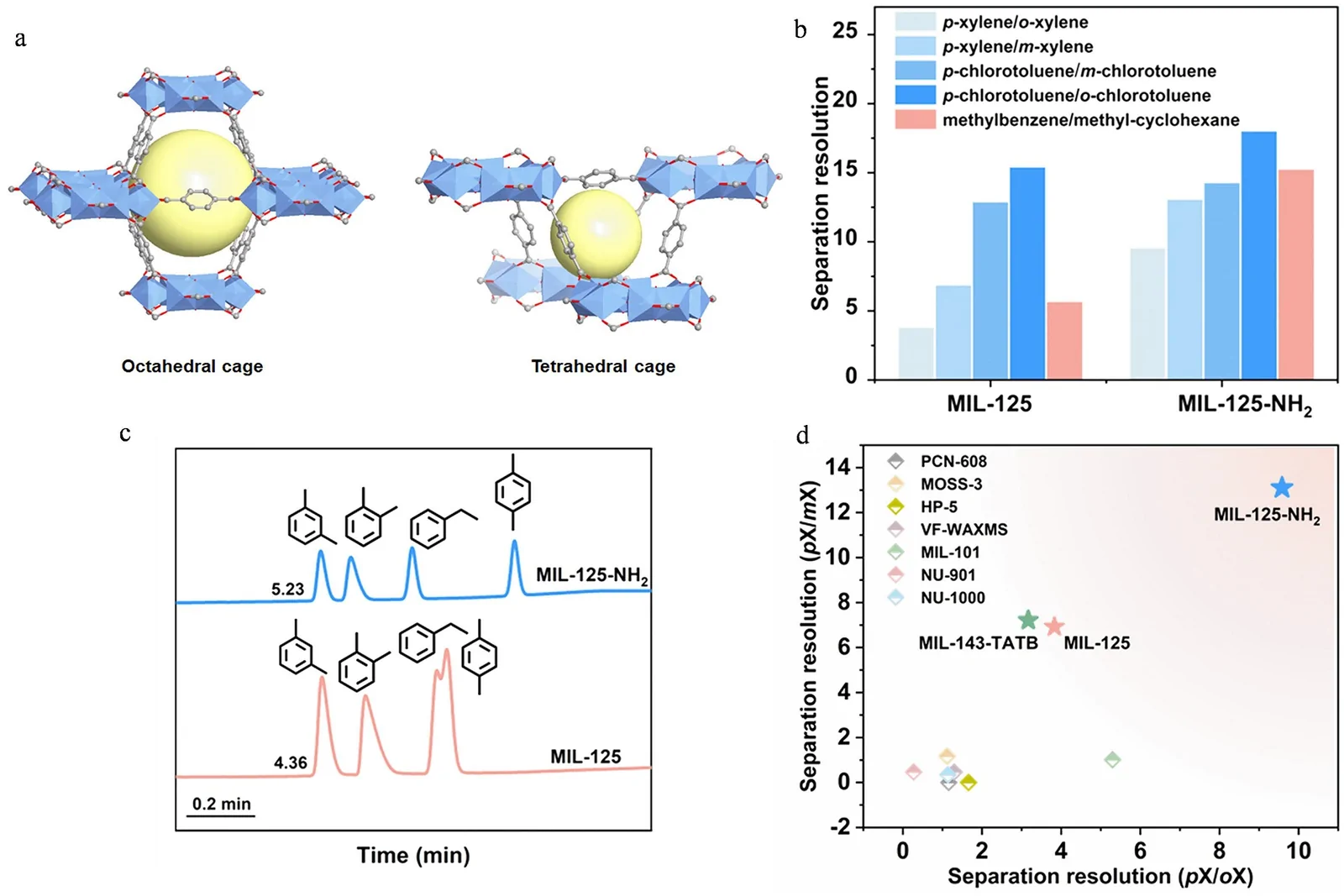
（a）MIL-125中八面体笼（1.2 nm）与四面体笼（0.6 nm）的结构示意图；（b）MIL-125与MIL-125-NH_2_涂覆的毛细管柱的分离度对比图；（c）MIL-125与MIL-125-NH_2_涂覆的毛细管柱的C_8_芳香族异构体分离图；（d）不同固定相对pX/mX、pX/oX的分离度对比图^［26］^

### 2.3 铝基MOF固定相

铝基金属有机骨架材料（Al-MOFs）在色谱领域的应用也较为广泛。除MOFs体系普遍具备的高比表面积和结构可设计性等优势外，典型Al-MOFs通常以一维铝氧链作为金属节点，构筑规则有序的一维孔道结构，有利于气体分子定向扩散与识别。此外，部分Al-MOF（如MIL-53）还具有“呼吸效应”，其孔道可在外界刺激下发生可逆柔性变形，从而增强对特定异构体的匹配能力与分离选择性。更为重要的是，与前述锆基和钛基材料相比，铝盐原料价格低廉，使得Al-MOFs在固定相制备中的整体成本显著降低，有利于其规模化制备与实际色谱应用。基于对色谱固定相产业化需求的综合考量，近两年来，本团队逐步将MOF固定相的研究重点由高稳定性但成本较高的体系，转向以铝基MOF为代表的低成本、高潜力材料体系，并围绕其结构调控、传质行为及分离性能开展了系统研究。2008年，Alaerts等^［52］^报道了MIL-53-Al在液相色谱中对C_8_芳香族异构体的选择性分离。具有一维菱形孔道（孔径约0.85 nm）的MIL-53-Al展现出对邻位烷基芳烃的优先吸附，并在脉冲色谱实验中成功实现EB、mX、pX和oX的依次洗脱。为探究其选择性机理，作者通过Rietveld精修确定了不同二甲苯分子在孔道中的吸附构型。结果表明，oX的两个甲基可同时与孔道中两个相对的羧酸根基团发生强相互作用，因此表现出最长时间的保留。相比之下，mX和pX仅能通过单一甲基与羧酸根作用，因而在MIL-53上吸附较弱、洗脱较早。该研究进一步比较了结构类似但金属节点不同的MIL-47对二甲苯的分离能力，发现二甲苯在两种材料上的出峰顺序完全不同，说明MIL-53中铝节点的独特电子环境与羧酸根的协同作用是其高邻位选择性的关键因素。

除MIL-53外，具有fym拓扑结构的Al-MOFs近年来逐渐成为分离领域的研究热点。该类材料通常由一维铝氧链与不同的V型有机配体自组装构筑而成，形成具有亚埃级孔径的一维限域孔道。值得注意的是，部分fym拓扑的Al-MOFs已被报道可实现大规模合成，为其在分离领域的实际应用奠定了基础。早在2018年，Wu等^［53］^就成功将fym拓扑的MIL-160制备成分离膜，用于分离二甲苯。MIL-160的孔道尺寸较小，只允许尺寸较小的pX通过而排除其他两种异构体，因此MIL-160分离膜表现出了良好的pX分离能力。Wang等^［2］^把同样的MIL-160制备成穿透柱，用于分离二甲苯蒸气。由于mX的尺寸与MIL-160孔道内吸附位点的位置最为匹配，该穿透柱表现出了良好的mX纯化能力。基于此，本课题组^［25］^选取了3种fym拓扑的Al-MOF，分别为CAU-10-H、MIL-160和CAU-10-NH_2_，制备气相色谱柱，研究了3种材料的GC分离能力。这3种材料分别采用V型配体间苯二甲酸（CAU-10-H）、2，5-呋喃二甲酸（MIL-160）和5-氨基间苯二甲酸（CAU-10-NH_2_）进行连接，形成了规整的一维纳米通道。实验表明，上述3种MOFs对二甲苯展现出截然不同的分离性能。其中，MIL-160固定相在程序升温条件下，对mX/pX、oX/pX和mX/oX的分离度分别高达16.28、19.00和4.55，超越了多种已报道MOFs材料以及商品化HP-5MS色谱柱。该工作通过一组结构明确的同拓扑Al-MOFs模型，证明了通过精准调控孔道尺寸与孔道表面化学性质，可调控fym拓扑Al-MOF固定相的分离性能。其中，MIL-160因其适中的孔径和呋喃环带来的适度相互作用，成功实现了热力学识别与动力学传质的最佳平衡，从而为二甲苯异构体的高效分离提供了一个优异的Al-MOF基固定相范例。最近，本团队^［24］^报道了一种新型的具有不对称一维曲折通道的铝基金属有机骨架材料Al-TCPB-Me_2_。与孔道曲折度较低的Al-TCPB相比，通道曲折度高的Al-TCPB-Me_2_可以基于尺寸筛分与孔内相互作用协同作用，表现出优异的二甲苯异构体分离性能（图8a~c）。Al-TCPB-Me_2_对异构体的分离度在报道时达到同类体系中的最高水平。该色谱柱同样表现出良好的分离稳定性和批次重现性。此外，Al-TCPB-Me_2_色谱柱可实现对高纯pX中痕量异构体杂质的精准定量分析（图8d），为工业级pX纯化过程中痕量杂质的高可靠性分析提供了一种切实可行的解决方案。

**图8:**
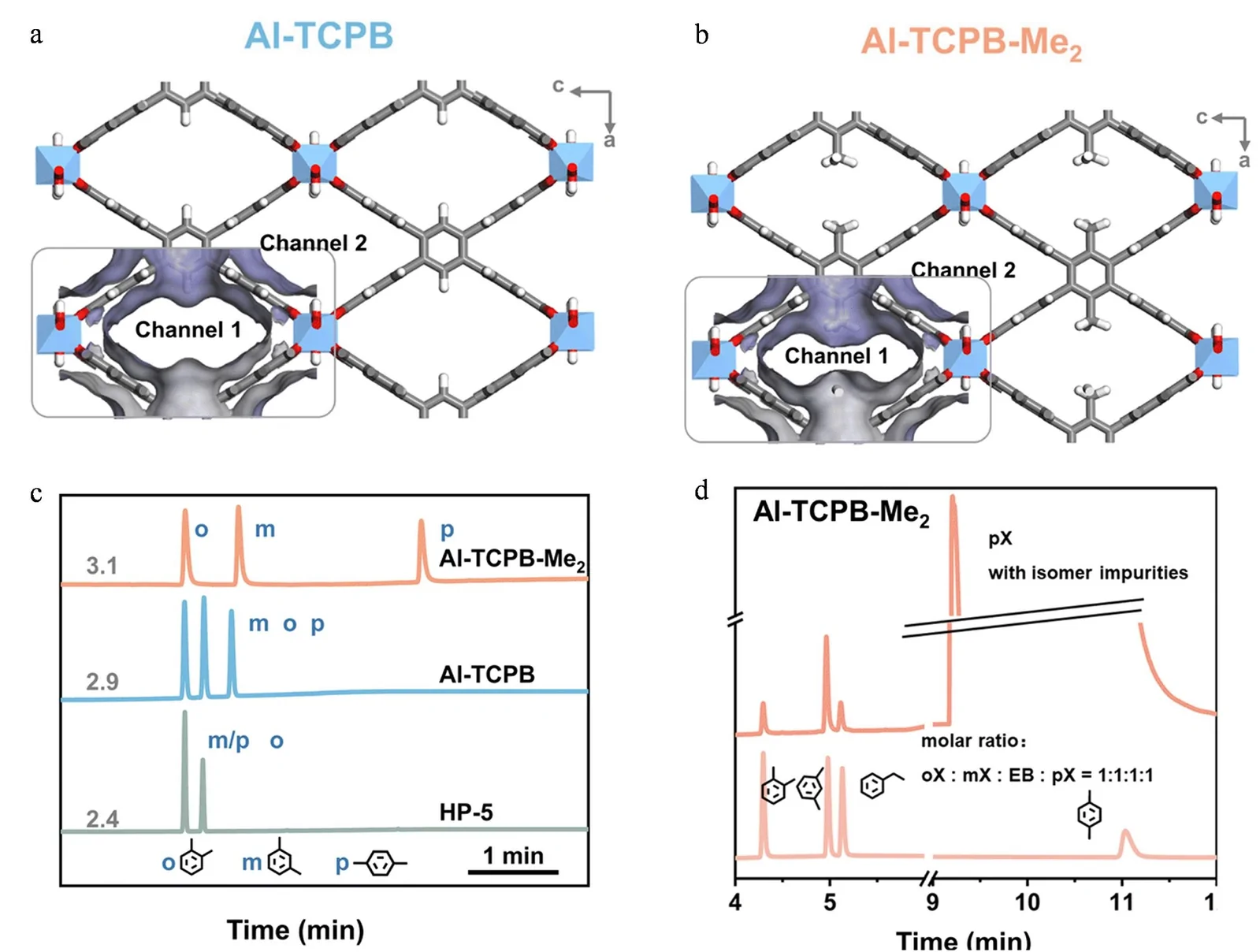
（a）Al-TCPB和（b）Al-TCPB-Me_2_的结构示意图；（c）二甲苯异构体气相色谱分离图和（d）含异构体杂质的对二甲苯气相色谱分离图^［24］^

### 2.4 铜基和锌基MOF固定相

铜基MOFs（Cu-MOFs）也是常见的经典MOFs材料，通常由铜离子或者铜簇和有机配体自组装而成。在色谱分离领域，Cu-MOFs也是一类应用较为广泛的固定相。2012年，Münch和Mertens^［54］^报道了基于HKUST-1涂层的毛细管GC柱用于二甲苯异构体的高效分离。HKUST-1是一类具有开放铜金属位点的经典Cu-MOFs，其孔径约为0.9 nm。研究团队采用循环涂覆的方法在毛细管内壁制备了约500 nm厚的HKUST-1薄膜，实现了二甲苯异构体的基线分离。尽管HKUST-1色谱柱表现出较好的二甲苯分离能力，但软酸（Cu）和硬碱（羧基）形成的配位键水稳定性较差，导致HKUST-1色谱柱在实际应用中面临结构稳定性不足的问题。铜和羧基形成的MOFs水稳定性有限，一直是制约该类材料广泛应用的关键挑战。为了解决以上问题，2025年，本团队^［19］^报道了一种超稳定的Cu_2_TBAPy纳米片（TBAPy=1，3，6，8-四（4-羧基苯）芘），该材料可以在较宽的pH范围（2~12）内稳定存在，甚至可以在沸水中稳定长达7天。Cu_2_TBAPy是一种二维铜羧酸MOF，其通过AB交错堆叠形成独特的“纳米信封结构”（图9a、9c），每个铜簇被两个疏水芘配体包覆，阻止了铜-羧基配位键被水进攻，因此具备了高的水稳定性。该材料的孔径为0.68~0.87 nm，与C_8_芳香族异构体分子尺寸（0.67~0.93 nm）高度匹配，同时保留了开放的铜位点及丰富的芳环相互作用位点。在色谱分离中，Cu_2_TBAPy色谱柱表现出优异的分离性能（图9b）。其中，mX/pX的*R*_s_高达18.2，发表时超过了所有已报道的气相色谱固定相。同时，由于该材料超高的稳定性，所涂覆的毛细管色谱柱可以在常规保存31个月后依旧保持原有的分离性能（图9d）。

**图9:**
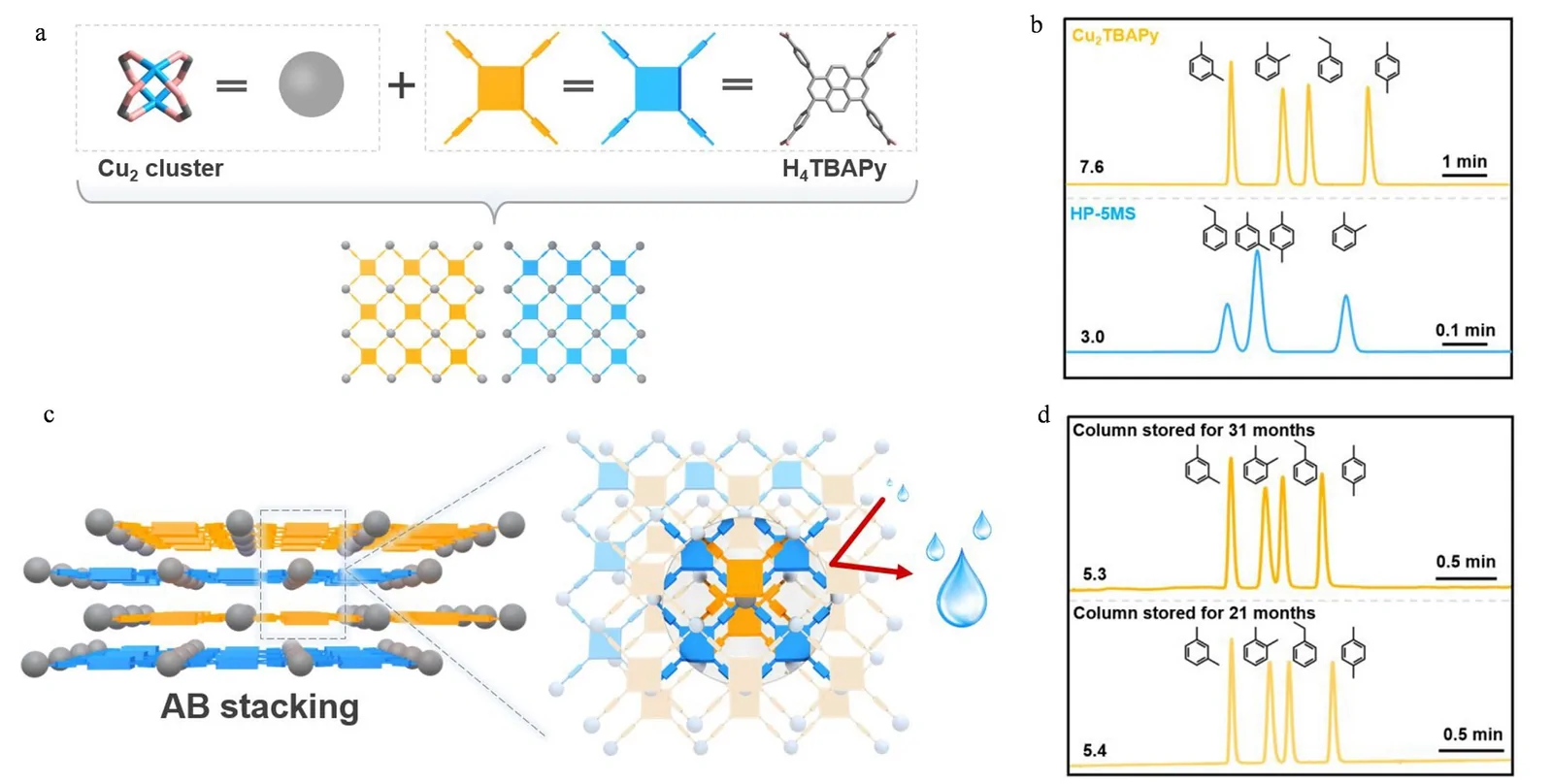
（a）Cu_2_TBAPy的合成示意图；（b）Cu_2_TBAPy色谱柱与商用HP-5MS色谱柱的C_8_芳香族异构体气相色谱分离图；（c）Cu_2_TBAPy的 AB 型堆叠组装；（d）常温条件下存放21个月和31个月的15 m Cu_2_TBAPy色谱柱对C_8_芳香族异构体的色谱分离图^［19］^

此外，该色谱柱也成功用于定量高纯pX中的痕量异构体杂质，杂质检测限低至1.33×10^‒12 ^g，为工业级pX纯化中痕量杂质的高精度检测提供了另一种可行的技术手段。

作为另一类常见的过渡金属，锌基MOFs（Zn-MOFs）因其丰富的结构多样性和可调孔道特性，也被广泛探索用于气相色谱分离应用。古志远教授^［55］^于2010年报道的研究中，考察了两种锌与BDC形成的MOFs材料（MOF-5与MOF-单斜）对二甲苯异构体及乙苯的吸附与分离性能。MOF-5具有由［Zn_4_O］^6+^次级建筑单元与对BDC连接构成的三维立方孔道（图10a），孔径约1.2 nm，具有非极性表面。MOF-5对二甲苯的分离主要受吸附平衡常数控制，其中，乙苯最先洗脱，3种二甲苯异构体则几乎同时流出（图10b）。相比之下，MOF-单斜具有三角形孔道（边缘长度约0.7 nm）和更高的极性，其分离行为受扩散控制。MOF-单斜对pX表现出优先吸附。气相色谱与吸附实验表明，MOF-单斜能基于孔径限制与扩散速率差异实现对pX的选择性分离，而MOF-5则主要通过热力学平衡常数差异分离乙苯与3种二甲苯异构体。Lin等^［46］^合成了一种具有分级孔结构的新型锌基多孔配位聚合物MCF-50。该材料具有三维非互穿网络结构，包含沿c轴方向的一维大六边形通道（有效直径约0.82~0.87 nm）（图10c）以及沿a轴和b轴方向的小四边形孔径（有效尺寸约为0.56 nm×0.68 nm）。这种层次孔系统兼具快速扩散通道和选择性吸附位点。该材料在气相色谱分离中表现出对二甲苯异构体的异常洗脱行为，其洗脱顺序为oX<mX<pX（图10d），明显不同于传统基于极性差异主导的分离机制。研究发现，小孔径作为主要吸附位点，可通过尺寸/形状匹配及骨架柔性区分不同异构体。pX因截面最小，可与孔径实现最佳匹配并引起骨架轻微收缩，从而获得最高的吸附焓。oX虽能完全进入孔道，但会引起孔径扩张，需消耗更多能量用于骨架形变。mX则因空间位阻仅部分进入孔道中，其吸附行为受主体-客体匹配能与骨架变形能共同调控，因此呈现中间出峰。

**图10:**
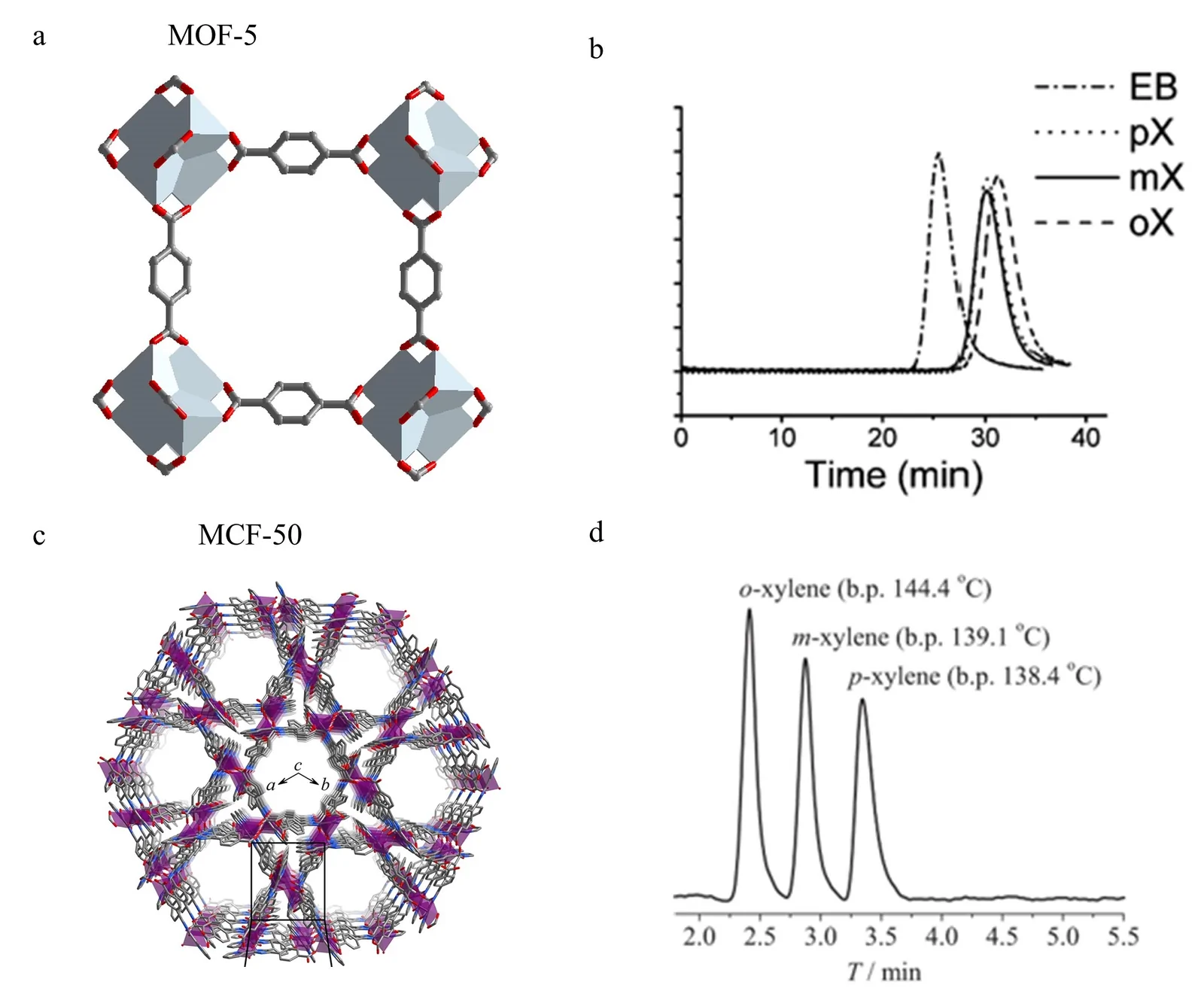
（a）MOF-5的结构示意图和（b）MOF-5色谱柱对C_8_芳香族异构体的气相色谱分离图^［55］^；（c）MCF-50结构示意图和（d）MCF-50色谱柱对二甲苯异构体的气相色谱分离图^［46］^

### 2.5 其他金属基MOF固定相

除了以上的锆基、钛基、铝基、铜基和锌基MOF固定相外，还有大量其他金属基的MOF也被报道用作GC固定相分离C_8_芳香族异构体，如铁、铬、铟、钒等。

本团队^［45］^以Fe‑MOF（MIL‑142‑BTB‑BDC）为平台，通过分别替换三齿配体和二齿配体，合成了MIL‑142‑TATB‑BDC与MIL‑142‑BTB‑BDC‑NH_2_。该系列材料具有超八面体空腔结构，其中三齿配体构成空腔壁，二齿配体形成连接空腔的三角形窗口（图11a、11b）。在二甲苯异构体分离中，MIL‑142‑BTB‑BDC与MIL‑142‑TATB‑BDC表现出相似的分离性能（图11c、11d），表明改变空腔壁配体对热力学相互作用的影响较小，仅略微降低了分析物的扩散动力学。而MIL‑142‑BTB‑BDC‑NH_2_因在空腔窗口引入氨基，增强了与分析物之间的热力学相互作用（如N-H…*π*作用），同时显著增加了传质阻力，导致峰展宽严重，分离性能显著下降（图11e）。该工作揭示了空腔窗口微环境对分离性能的主导作用。古志远教授^［56］^首次报道了以铬基MOF（MIL‑101）作为固定相的高效气相色谱毛细管柱。MIL‑101具有笼状大孔（2.9~3.4 nm）及配位不饱和位点，通过动态涂覆法制备的毛细管柱在等温条件下（160 ℃）于1.6 min内实现了pX、mX、oX及EB的基线分离，理论塔板数高达3 800 plates/m。其分离机制不仅依赖于主‑客体相互作用，更与MIL‑101中配位不饱和位点及其适宜极性密切相关。通过后合成修饰将吡啶接枝于配位不饱和位点后，分离选择性下降，进一步证实了该类位点在分离中的关键作用。Li等^［57］^合成了一系列具有一维方形孔道的MFM‑300（M）材料（M=In、V、Fe和Al），并将这些材料作为液相色谱固定相研究了它们的C_8_芳香族异构体分离性能。由于金属节点的不同，这一系列MFM-300（M）的孔径为0.65~0.74 nm。其中，MFM‑300（In）、MFM‑300（V）及MFM‑300（Fe）均表现出相同的洗脱顺序，即pX先于oX出峰，最后为mX。MFM‑300（V）和MFM‑300（Fe）的对‑间二甲苯选择因子分别为15和18。MFM‑300（Al）因孔径过小，分离性能较差。通过串联MFM‑300（In）与MFM‑300（V）色谱柱，实现了3种异构体的近乎完全分离。结合同步辐射X射线衍射与太赫兹光谱分析，揭示了孔道内主-客体*π-π*相互作用及与羧基氧原子的范德华作用共同主导了对mX的选择性吸附。

**图11:**
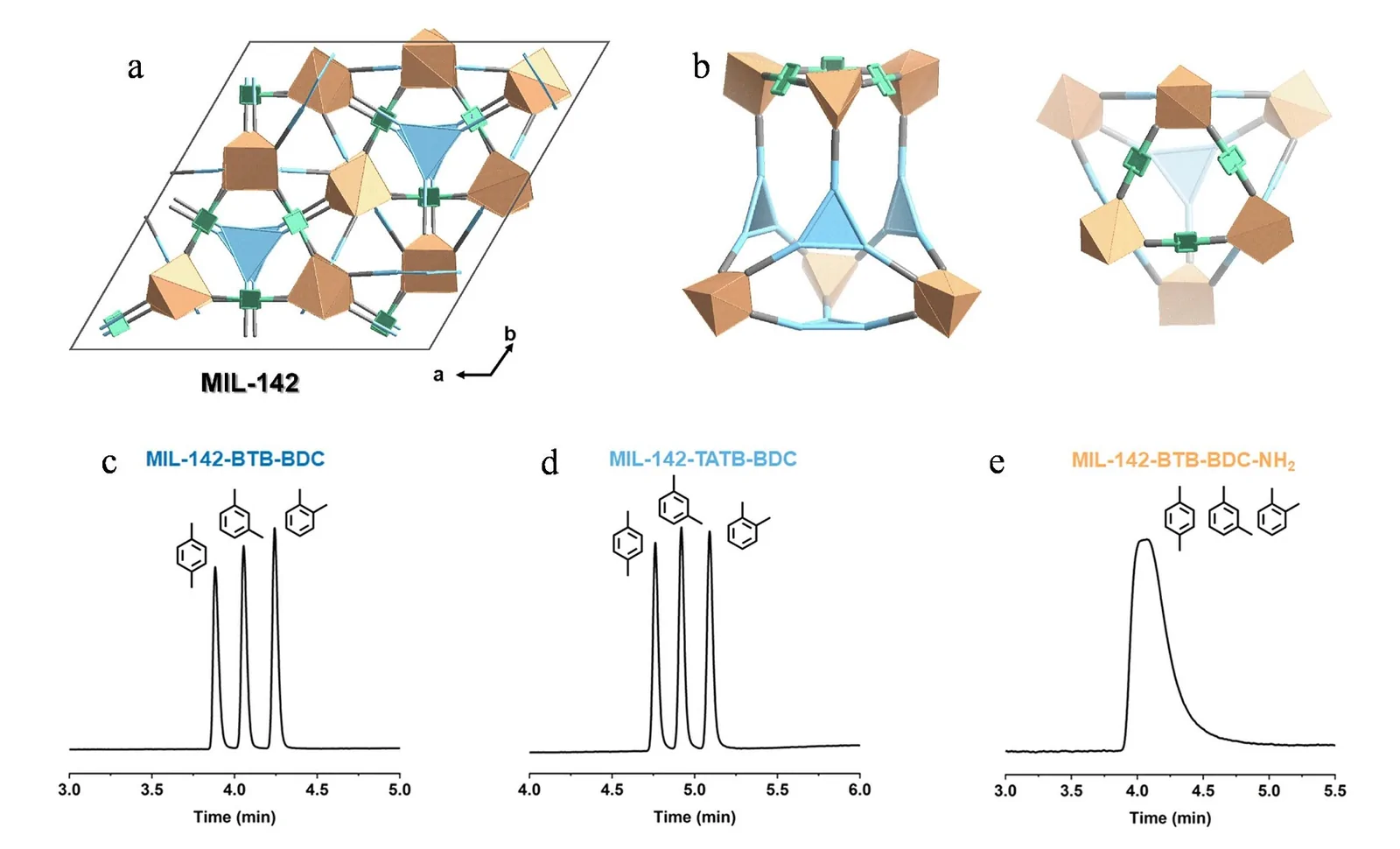
（a）MIL-142的结构示意图和（b）超八面体沿不同方向的结构示意图；（c）MIL-142-BTB-BDC、（d）MIL-142-TATB-BDC和（e）MIL-142-BTB-BDC-NH_2_色谱柱对二甲苯异构体的气相色谱分离图^［45］^

需要指出的是，上述内容仅选取了若干具有代表性的金属基MOF材料进行综述，用以阐明不同金属节点与孔结构调控策略在C_8_芳香族异构体分离中的作用机理。事实上，除上述体系外，仍有多种MOF材料（如MOF-74、NU-1000等）被报道可实现对C_8_芳香族异构体的有效分离^［58-61］^，相关研究在此不再一一展开。

## 3 MOF色谱柱的表面修饰与构筑策略

在色谱分离体系中，色谱柱的整体结构与内壁表面状态直接决定了固定相的分布形态、传质行为以及分离性能的稳定性。对于MOF基色谱柱而言，其分离性能不仅取决于MOF材料本身的孔道结构与主-客体相互作用，更在很大程度上受制于色谱柱内壁表面修饰方式及固定相-柱壁界面的构筑质量。如果缺乏合理的柱内表面调控，即使具有优异分离性能的MOF材料，也难以在色谱柱中实现均匀、稳定且可重复的分离效果。

从工程实现角度看，MOF色谱柱的构建涉及两个层级的问题：一是色谱柱内壁的表面修饰与活化，以构建适于固定相负载的界面；二是在此基础上，通过合适的方法将MOF固定相均匀、牢固地引入色谱柱内。因此，柱内表面修饰应作为MOF色谱柱构筑的前提步骤，而固定相的涂覆或生长方式则是对该界面设计的具体实现。

### 3.1 色谱柱的表面修饰

在MOF色谱柱的构建过程中，柱内壁的化学修饰是实现固定相稳定负载与可重复分离性能的关键前提。常用的毛细管气相色谱柱内壁为石英，其表面经活化处理后可暴露大量硅醇基（-Si-OH），为后续硅烷化反应提供反应位点。基于不同构筑策略的需求，通常可引入氨基或羧基官能化内壁来调控固定相-柱壁之间的界面相互作用。

在氨基化修饰中，最常用的试剂为3-氨丙基三乙氧基硅烷（APTES）。APTES可通过其乙氧基与石英内壁表面的-Si-OH发生缩合反应，在柱内壁形成稳定的Si-O-Si键，同时在界面层暴露出末端-NH_2_基团。该氨基化内壁一方面能够通过氢键或静电作用增强MOF颗粒与柱壁之间的附着力，另一方面在原位生长法中可作为金属离子的配位位点，促进MOF在柱内壁上的成核与定向生长，从而获得结合牢固、分布均匀的固定相涂层。本团队^［62］^研究了修饰APTES和未修饰APTES的毛细管柱，涂覆相同的材料之后的色谱分离结果。未修饰APTES的毛细管柱在涂覆材料之后表现出来的分离能力明显较差。

相比氨基化修饰，羧基化内壁修饰更有利于与金属节点形成强相互作用，尤其适用于采用羧酸配体构筑的MOFs。该策略通常通过在柱内壁引入含羧基的硅烷偶联剂（如羧乙基三乙氧基硅烷或经后续氧化得到的羧基硅烷层），在石英表面构建暴露-COOH官能团的界面。羧基不仅能够与金属离子形成配位或静电相互作用，还可在原位生长过程中为MOF晶体提供稳定的锚定位点，有效抑制涂层在载气冲刷与温度循环条件下的脱落。Böhle与Mertens^［63］^采用羧基化界面修饰策略，在毛细管内壁构建了羧基终止的自组装单分子层，并通过循环沉积法将［Cu_2_（bdc）_2_（dabco）］ MOF（dabco=1，4diazabicyclo［2.2.2］octane）原位生长并锚定于表面。该策略利用羧基与MOF中铜节点的强配位作用，有效增强了涂层与柱壁的结合强度及热稳定性，实现了对C_1_-C_7_烷烃混合物的基线分离。

总之，氨基化或羧基化内壁均可显著改善MOF颗粒在柱内的润湿性与分散性，减少局部堆积和非均匀涂覆现象，有利于形成薄而连续的固定相层，从而降低传质阻力并提升柱效。通过合理选择内壁官能团类型，可实现对固定相附着方式与界面稳定性的针对性调控。

### 3.2 色谱柱的构筑

目前，MOF色谱固定相的构筑方法主要包括原位生长法、静态涂覆法和动态涂覆法，不同方法在涂层均匀性、结合强度、操作复杂度及适用材料体系等方面各具特点。

原位生长法是指在色谱柱内壁表面直接诱导MOFs的成核与生长，使其在预定位置形成均匀且稳定的固定相涂层。该方法通常通过调控前驱体溶液组成、反应温度及时间，实现MOF在毛细管内壁的可控生长。该方法通过控制化学反应条件，使目标材料在预定位置自发生长并形成均匀、稳定的涂层或固定相，从而实现对色谱分离性能的优化。如上文提到的Münch等^［54］^即采用了循环层沉积方法在毛细管内壁原位生长了HKUST-1涂层GC固定相，并成功实现了苯、甲苯、乙苯以及邻、间、对二甲苯等芳烃混合物的基线分离。

静态涂覆法最初是由Golay提出的^［64］^，这是目前生产高性能色谱柱最常用、最可靠的方法。静态涂覆法是指将固定相溶解在合适的挥发性溶剂中，配制成浓度精确的稀溶液，并将配好的溶液通过压力灌入毛细管中，然后封住一端，待溶剂缓慢蒸发完全之后，通过缓慢升温（通常在有惰性气体保护下），使固定相初步固化，形成稳定的涂层。该方法的优点是可以确保涂层厚度的均匀性，同时还可以根据固定相溶液的体积和浓度很容易地计算出β值和液膜厚度。Sun等^［65］^采用静态涂覆法制备两亲性柱［6］芳烃固定相，实现了芳香胺异构体的高效分离。

与静态涂覆法相比，动态涂覆法因操作简便、制备周期短而在色谱领域得到广泛应用。动态涂覆法是由Dijkstra and De Goey首次提出的^［64］^，动态涂覆的核心原理是通过惰性气体（如氮气）推动含有固定相的溶液在毛细管柱内流动，溶液在流动过程中与毛细管内壁接触，部分固定相分子吸附或沉积在内壁上，形成一层液膜。随着溶液的持续流动，液膜逐渐覆盖整个内壁，完成涂层制备。动态涂覆法是本团队最常用的涂覆方法。比如，最近本课题组^［48］^采用此方法，将3种具有相同frl拓扑结构的铝基MOF（Al-L-H、Al-L-OMe和Al-L-OCp，OCp=cyclopentyloxy）成功涂覆于毛细管内壁，制备出了高性能色谱柱。二甲苯异构体分离实验结果表明，Al-L-OMe固定相展现出优异的分离能力，其单一扩散通道与适中的主-客体相互作用是实现基线分离的关键。其中Al-L-OMe固定相对pX与oX的分离度高达13.74，显著优于多数已报道的MOF基固定相及商用常规色谱柱。为了更清晰地呈现这一结果及相关对比数据，表2汇总了不同固定相对二甲苯异构体的分离度数值。

**表2:** 二甲苯异构体在不同色谱固定相中的分离度

Material	*R*_s_ （mX/oX）	*R*_s_ （mX/pX）	*R*_s_ （oX/pX）	Ref.
PCN-608-N	1.16	0	1.16	［^27^］
NU-1000-N	0.34	0.44	1.05	［^27^］
Cu_2_TBAPy	5.18	18.2	8.44	［^19^］
MIL-125-NH_2_	1.44	13.10	9.85	［^26^］
MIL-142-BTB-BDC	3.03	2.78	5.75	［^45^］
MIL-160	4.55	16.28	19.00	［^25^］
CAU-10-H	0.87	12.26	10.55	［^25^］
NU-901	1.07	1.15	1.08	［^23^］
MCF-50	1.9	1.65	3.25	［^46^］
MOSS	1.12	1.06	1.06	［^29^］
Zr-BTB	/	3.27	1.67	［^47^］
Al-TCPB-Me_2_	7.4	24.9	31.1	［^24^］
Al-L-OMe	7.35	6.38	13.74	［^48^］
CTMOF-2	/	1.29	5.96	［^30^］
HP-5MS	/	0.15	4.84	［^19^］
VF-WAXMS	/	0.47	1.3	［^19^］

总体而言，不同固定相构筑方法在MOF色谱柱性能调控中各具优势。通过合理选择构筑策略，并结合孔道工程与表面化学修饰，有望进一步提升MOF固定相在复杂异构体体系中的分离能力与应用可靠性。

## 4 MOFs固定相的应用与发展趋势

近年来，MOFs作为一类具有可设计孔道结构和多样化主-客体相互作用的新型多孔材料，为突破C_8_芳香族异构体分离瓶颈提供了新的固定相平台。围绕MOF固定相在C_8_芳香族异构体分离中的应用研究，主要向减少共流出提升分离度、实现痕量目标物检测以及降低成本等方向发展，逐步展现出从方法学优化向实际分析应用拓展的潜力。

首先，在MOF固定相用于C_8_芳香族异构体分离的研究中，抑制共流出、提升异构体分离度已成为最核心的发展方向之一。由于邻、间、对二甲苯在分子尺寸、构型及极性上的差异极其微弱，传统商品固定相往往难以提供足够的选择性，mX与pX等关键异构体在实际分析中极易发生共流出，严重制约了分离效率与分析可靠性。将MOFs引入色谱固定相体系，通过结构可设计的多孔骨架放大异构体之间的微小差异。得益于可精确调控的孔径尺寸、孔道形状及孔内化学环境，MOF固定相在分离过程中能够协同引入尺寸筛分、形状选择性、*π-π*相互作用及扩散动力学差异等多种分离机制，从而显著提升C_8_芳香族异构体之间的有效分离度。近年来，该策略^［25］^已在多个体系中取得突破性进展，例如mX与pX的分离度由商业柱HP-5MS上的几乎为0，被本团队大幅提升至MIL-160色谱柱上的16.28，这充分表明MOF固定相在解决二甲苯异构体共流出问题方面展现出独特优势和广阔应用前景。

第二，高纯样品中痕量杂质的准确检测是MOF固定相设计与应用中的一项核心发展方向。高纯乃至超纯化学品是高端制造、精细化工及先进材料领域的重要基础原料，其性能往往对痕量杂质高度敏感，即使极低含量的异构体或结构相似杂质，也可能对产品质量和应用性能产生显著影响。因此，实现对高纯化学品中痕量杂质的可靠分离与准确定量，具有重要的实际意义和应用价值。在二甲苯体系中，由于各异构体在分子结构和理化性质上高度相似，传统固定相往往难以在高分离度与高柱容量之间取得平衡；主成分峰易严重掩盖痕量杂质，或使杂质信号淹没于主峰拖尾中，进而导致定量结果偏差甚至误判。针对这一分析难题，MOF固定相通过构建具有特定孔径与表面化学环境的有序孔道体系，可在保持主成分有效洗脱的同时，对痕量异构体杂质产生差异化吸附与传质行为，从而实现杂质检测窗口与主成分信号的有效解耦。比如，本团队发展的Al-TCPB-Me_2_色谱柱成功实现了对二甲苯异构体的高效分离与痕量杂质检测^［24］^。实验表明，该柱在痕量分析方面表现出极高的灵敏度，对oX、mX、pX及EB的检测限（LOD，*S/N*=3）分别为（2.59±0.22）×10^‒12^ g、（3.11±0.22）×10^‒12^ g、（15.39±1.99）×10^‒12^ g和（3.08±0.13）×10^‒12^ g。尤为重要的是，在实际高纯pX样品分析中，该柱能够在高浓度主峰存在的条件下，可靠定量浓度低至（2.00±0.30）×10^‒4^（oX）、（8.00±0.10）×10^‒4^（mX）、（2.00±0.20）×10^‒4^（EB）的痕量异构体杂质，充分体现了MOF固定相在复杂基质中开展超痕量分析的应用潜力。

最后，从实际应用角度出发，色谱固定相的发展正逐步朝着低合成成本、低储存成本以及长使用寿命的方向演进。早期用于C_8_芳香族异构体分离研究的MOF固定相，如MOF-5和HKUST-1等，虽在一定程度上验证了MOF孔道结构在异构体分离中的可行性，但其金属节点与羧基配体之间形成的配位键水稳定性较差，对储存环境和使用条件要求较高，易发生结构降解，导致色谱柱寿命有限，从而在很大程度上制约了其进一步的实际应用。为克服这一瓶颈，研究者逐渐将注意力转向具有更强配位键稳定性的MOF体系，典型代表为以高价金属节点（如Zr）与羧基配体构筑的骨架结构。这类材料通常表现出优异的水热稳定性和化学惰性，显著延长了色谱柱的使用寿命。基于此，本团队^［19，27］^陆续报道了如PCN-608和Cu_2_-TBAPy等一系列高稳定性MOF固定相，其色谱柱寿命可达半年甚至两年以上，为MOF固定相的长期稳定运行奠定了基础。在此基础上，近年来相关研究进一步聚焦于兼顾稳定性与经济性的MOF固定相体系，开始探索由廉价金属节点与易得有机配体构筑的MOF材料。例如，以铝为金属节点的MIL-160等Al基MOF，不仅原料成本低、合成条件温和，而且在保持良好结构稳定性的同时，仍能提供适宜的孔道环境用于C_8_芳香族异构体的高效分离。此类材料的出现，标志着MOF固定相研究正由“性能验证型”向“应用导向型”转变，为其在工业分析与常规检测中的规模化应用提供了更具现实意义的解决方案。

## 5 总结与展望

二甲苯异构体与乙基苯由于分子组成相同、分子质量一致且理化性质高度相似，长期以来被视为色谱分离领域中的经典难题之一。MOFs凭借其高度有序且可精确调控的孔道结构、丰富且可设计的孔内化学环境，以及多样化的主客体相互作用机制，为解决二甲苯异构体与乙基苯高效分离这一难题提供了全新的材料体系与研究范式。本文系统综述了MOFs作为色谱固定相在C_8_芳香族异构体分离领域中的研究进展。首先，概述了C_8_芳香族异构体分离的基本理论基础，重点阐明了尺寸效应、形状选择性、孔内相互作用及扩散动力学等因素在分离过程中的作用机理。随后，从不同金属节点（如Cu、Zn、Al、Zr、Ti等）构筑的代表性MOF材料出发，系统总结了其在孔径尺寸调控、孔道形貌设计、表面化学环境构建以及传质行为调节等方面对C_8_芳香族异构体分离性能的影响规律。在此基础上，进一步介绍了色谱柱内壁表面修饰方法及MOF色谱柱的构筑策略，重点讨论了不同构筑方式对固定相均匀性、稳定性及分离性能的影响。最后，结合具体研究实例，总结了MOF固定相在提升分离度、实现痕量杂质检测以及降低成本等方面的应用进展和发展趋势。

尽管MOF固定相在C_8_芳香族异构体分离领域已展现出显著优势，但其从实验室研究向广泛实际应用的转化仍面临关键科学与工程问题。展望未来，MOF固定相在C_8_芳香族异构体分离领域的发展可从以下几个方向进一步推进：（1）在材料层面，稳定性与批次重现性仍是阻碍MOF固定相进一步推广的关键因素。未来研究亟须在保证分离性能的同时，实现MOF固定相在稳定性、批次一致性与使用寿命之间的综合平衡，并建立面向工业复杂工况（如高温高压、含水气氛）的系统性稳定评价体系，以指导其实际应用与产业化。（2）在色谱应用层面，C_8_芳香族异构体分离的核心挑战已逐渐从“是否能够分开”转向“是否能够在复杂条件下稳定、可靠地分开”。特别是在高纯样品分析中，主成分与痕量杂质之间巨大的浓度差异，极易导致杂质信号被掩盖或淹没于主峰拖尾之中。未来MOF固定相的设计不仅需要进一步提升分离度，更应关注杂质检测窗口与主成分信号的有效解耦，实现对痕量异构体杂质的准确识别与定量，以满足精细化工和高端制造领域对分析可靠性的更高要求。（3）此外，随着现场检测与实时分析需求的不断增长，色谱技术正朝着微型化、集成化与低功耗方向发展。MOF固定相凭借其可设计孔道结构和高比表面积，在微型色谱系统中展现出独特优势。然而，如何实现MOF材料在微尺度通道内的均匀负载、稳固固定及高效传质，仍面临诸多工程挑战。未来有必要将MOF固定相的材料设计与MEMS制造工艺深度融合，探索适用于微型色谱柱的新型构筑与固定化策略，从而推动其在便携化、现场快速检测中的实际应用。（4）从色谱机理方面，目前的大多数研究仍基于晶体结构的静态描述，但色谱分离是高温下的动态过程。应重点关注MOF骨架在载气流动下的微小变化如何影响异构体的扩散能垒，加深对色谱分离机理的研究。（5）从更长远的角度看，引入计算模拟、分子动力学及机器学习等数据驱动方法，将为MOF固定相的理性设计提供新的技术路径。通过建立MOF结构参数、孔内相互作用特征与色谱分离性能之间的定量关联模型，加速材料筛选与结构优化过程，减少试错成本，并推动MOF固定相开发由经验导向向模型驱动转变。总体而言，随着材料设计理念、色谱构筑技术与数据分析方法的不断融合，MOF固定相有望在C_8_芳香族异构体分离及相关复杂体系分析中展现更广阔的应用前景。
